# Abstracts from the 9th International Conference for Healthcare and Medical Students (ICHAMS): Dublin, Ireland. 6-8 February 2020

**DOI:** 10.1186/s12919-020-00185-1

**Published:** 2020-04-30

## Plenary session

### A1 Glycosylation and proteolytic cleavage of α-dystroglycan (α-DG) in thrombin activated platelets

#### Austin B. Auyeung^1^, Wenjing Ma^2^, Reid Gallant^2^, Yiming Wang^2,3^, Heyu Ni^2,4^

##### ^1^School of Medicine, Royal College of Surgeons in Ireland, Dublin, Ireland; ^2^Keenan Research Centre for Biomedical Science, St. Michael’s Hospital, Toronto, Canada; ^3^Clinical and Metabolic Genetics, The Hospital for Sick Children, Toronto, Canada; ^4^Canadian Blood Services, Toronto, Canada

###### **Correspondence:** Austin B. Auyeung

**Introduction**

Αlpha-dystroglycan (α-DG) is a dystrophin-associated cell membrane glycoprotein and a vital component of the dystrophin complex, which plays a crucial role in cell adhesion to the extracellular matrix (ECM) in myocytes. Our group has shown that α-DG and likely other components of the dystrophin complex are expressed on the platelet surface. Targeting the dystroglycan complex by blocking α-DG inhibits platelet adhesion, aggregation, and thrombus formation, possibly through formation of a α-DG-fibronectin-αIIbβ3 complex. Proteolytic cleavage and glycosylation of α-DG modulate its adhesion to laminin and fibronectin in ECM. However, how these post-translational modifications respond and contribute to platelet activation, adhesion and aggregation have never been investigated.

**Methods**

Whole blood from mice or venous blood from healthy adult volunteers were centrifuged and the top 2/3 of platelet-rich plasma (PRP) was collected. PRP was activated with 1 U/ml thrombin or left unactivated for 5 min at 37°C. We used two monoclonal anti-α-DG antibodies IIH6C4 and VIA-4 to detect α-DG cleavage (band size at ~100kD after cleavage while the intact protein is ~150kD) and glycosylation (with glycan-dependent antigen recognition antibodies, stronger binding signal reflects increased glycosylation) in resting and thrombin activated mouse/human platelets using flow cytometry and western blot.

**Results**

Compared to resting platelets, thrombin activated platelets have increased IIH6C4/VIA4 surface and whole cell lysate binding, as measured by flow cytometry and western blot, respectively. α-DG may be stored in α-granules and translocated to the cell surface and/or have its N-terminal removed and/or be glycosylated upon thrombin activation.

**Discussion**

It has been reported that N-terminal removed and glycosylated forms of α-DG have a much higher ligand binding affinity. Therefore, after thrombin activation, α-DG may engage more/stronger fibronetin-αIIbβ3 binding to enhance platelet-platelet interaction/platelet- ECM adhesion through its post-translational modification. These may serve as novel targets for the treatment of thrombotic disorders.

### A2 Evaluation of efficacy of mannitol vs. hypertonic saline for reducing intracranial pressure in patients with severe traumatic brain injury: A network meta-analysis

#### Radhe Shah^1^, Ayush Thakkar^2^, Pooja Rangwala^3^, Devang Rana^1^

##### ^1^Smt. NHL Municipal Medical College, Ahmedabad, India_;_^2^GCS Medical College, Hospital and Research Centre, Ahmedabad, India; ^3^AMC MET Medical College, Ahmedabad, India

###### **Correspondence:** Radhe Shah

**Introduction**

Mannitol is used as the gold standard and Hypertonic Saline(HTS) as the second-line drug for hyperosmolar therapy to reduce Intracranial pressure(ICP) in patients of severe traumatic brain injury (STBI). Recent times have shown an increased interest in replacing Mannitol with HTS as the first-line drug. Individual trials comparing the two show certain discrepancies in the results and this meta-analysis aims at eliminating the same.

**Methods**

PubMed, Cochrane, Google Scholar, MeSH, and Embase databases were searched until 8^th^ Feb 2019. RCTs and prospective studies, following the PRISMA guidelines and inclusion criteria where Mannitol or HTS were administered for increased ICP in STBI (Glasgow Coma Scale: 3-8) were included. The primary outcome was the change in ICP 30, 60, 90, and 120 minutes after drug administration. For the measurement of treatment effect RevMan 5.3 version software by Cochrane Database was utilized to calculate Odd’s ratio. A Random and Fixed effect model was applied to calculate the standardised mean difference of change between groups. P value less than 0.05 was considered as a statistically significant value. The I^2^ was used to measure the heterogeneity between studies and a value > 30.0 was considered to reflect heterogeneity.

**Results**

A total of 8 studies with 276 patients met the inclusion criteria**.** The mean ICP reduction after 30 minutes of drug administration in HTS group was 7.69±3.18(95%CI=4.7508- 10.6464) and for mannitol group was 6.28±4.92(95%CI=1.7291-10.8452). Test for heterogeneity, I^2^=0.00%, p=0.9380. The mean ICP reduction after 120 minutes of drug administration in HTS group was 8.31±2.91(95%CI=5.62-11) and for mannitol group was 7.22±3.74(95%CI=2.57-11.87). Test for heterogeneity, I^2^=32.16%, p=0.2070. No statistical difference between the two drugs at 30minutes(p=0.677), 60minutes(p=0.639) and 120minutes(p=0.367) after administration was observed.

**Discussion**

Thus, Mannitol and HTS can be used interchangeably to reduce ICP in patients of STBI in view of no significant difference in efficacy.

## Oral session

### O1 Evaluating the growth of melanoma cells in 3D in vitro using collagen-based scaffolds

#### Evgeniia Mustafaeva^1^, John Nolan^2^, Olga Piskareva^3,4^

##### ^1^School of Medicine, Royal College of Surgeons in Ireland, Dublin, Ireland; ^2^School Of Pharmacy and Biomolecular Sciences, Royal College of Surgeons, Dublin, Ireland; ^3^School of Pharmacy and Regenerative Medicine, Royal College of Surgeons, Dublin, Ireland; ^4^Department of Anatomy and Regenerative Medicine, Royal College of Surgeons, Dublin, Ireland

###### **Correspondence:** Evgeniia Mustafaeva

Melanoma, a cancer of melanocytes, is one of the most common cancers in the world. In living tissues, it grows surrounded by a 3D microenvironment, which provides physical support and determines disease progression and prognosis. The aim of this project was to determine how different melanoma cell lines M14 and SK-MEL-28 behave, grow and expand in 3D in vitro models using collagen-based scaffolds. Collagen scaffolds contained either chondroitin to mimic skin tissue, hyaluronic acid or nano-hydroxyapatite to mimic bone tissue.

All tested scaffolds were populated with melanoma M14 and SK-MEL-28 cells that were left to grow for 28 days. Scaffold infiltrations by cells were assessed on day 1, 7, 14, 21 and 28. Populated scaffolds were then processed with a tissue processor, embedded in paraffin wax, sliced up with a microtome and stained with H&E followed by bright field microscopy. The images were put together in chronological order to see the growth progression.

Both SK-MEL-28 and M14 cell lines have demonstrated most penetration when grown on chondroitin scaffolds, followed by hyaluronic acid scaffolds. Cells grown on nano-hydroxyapatite scaffolds showed the least colonisation and tended to accumulate around the edges of the scaffold. M14 cells growing on chondroitin scaffolds demonstrated rapid infiltration when compared with SK-MEL28 cells probably due to intrinsic aggressive growth properties.

In conclusion, we have demonstrated for the first time the ability of collagen-based scaffolds to support melanoma cell growth and colonisation. Our findings can aid the development of a 3D in vitro scaffold-based platform to study melanoma biology.

### O2 Exploring new mechanisms for HIF-2 activation

#### William B. Sherwood, Brian M. Ortmann, Guinevere L. Grice, James A. Nathan

##### Cambridge Institute of Medical Research, Keith Peters Building, Department of Medicine, University of Cambridge, Cambridge, England

###### **Correspondence:** William B. Sherwood

**Introduction**

Hypoxia Inducible transcription Factors (HIFs) are central to the cellular response to oxygen availability, activating a myriad of genes to ensure cell survival. However, inappropriate HIF activation promotes tumour formation and alters immunity. Whether HIF-2a, the main driver for solid-organ tumours, is regulated distinctly from HIF-1a, the constitutively expressed form, is debated. Here, we use CRISPR/Cas9 genome-wide forward genetic screens to identify regulators of HIF-2a turnover in normoxia.

**Methods**

Ten genes were identified for validation from this HIF-2a activation screen. Genetic depletion of known HIF regulators (VHL, PHD2, and OGDH), or chemical inhibition of the HIF- 2a degradation pathway, activated HIF-2a as expected. Three CRISPR sgRNAs, for each candidate gene, were designed and lentivirally transduced into HIF-2a GFP reporter cells. GFP fluorescence was monitored by flow cytometry, and immunoblotting was performed for the endogenous proteins. As a complimentary validation strategy, the HIF-2a activation screen was optimised and repeated.

**Results**

Both screens identified high confidence hits involving the canonical regulation of HIF- 1a and HIF-2a, validating the approach. Genes required for the OGDHc were also identified and validated in unrelated cells to the screen, consistent with OGDHc function regulating both HIF- 1a and HIF-2a. Several other top candidate genes were selected for further investigation. Tectonin Beta-Propeller Repeat Containing 1 (TECPR1) and Meiotic Recombination 11 homolog A (MRE11a) knockdowns initially showed HIF reporter activation after 10 days. However, this was not reproduced in independent cell lines.

**Discussion**

The CRISPR-Cas9 screens reproducibly identified known regulators of HIF-a, and showed that the OGDHc also regulated the HIF-2a isoform. Currently, no genes that regulate HIF-2a distinctly from HIF-1a have been identified. While further optimisation of the HIF-2a screening approach is required, these findings are consistent with the HIF-a isoforms being post-translationally regulated in the same manner.

### O3 Is the initial improvement in cardiovascular morbidity sustained at 2 years following bariatric surgery?

#### Kwun Hang Aaron Ho^1^, Colm J. O’Boyle^2^, Hayder Shabana^2^

##### ^1^School of Medicine, University College Cork, Cork, Ireland; ^2^Department of Surgery, Bons Secours Hospital, Cork, Ireland

###### **Correspondence:** Kwun Hang Aaron Ho

**Introduction**

Although morbid obesity is strongly associated with hypertension, relatively little research has been performed to evaluate the long-term antihypertensive impact of bariatric surgery. We aim to assess the impact of bariatric surgery on hypertension and antihypertensive usage over a 2-year period.

**Methods**

This study was a retrospective review of 618 patients undergoing laparoscopic sleeve gastrectomy (LSG) and gastric bypass (LGBYP) under the care of a single Consultant Surgeon between January 2009 and June 2019. Weight, height, body mass index (BMI), systolic blood pressure (SBP), diastolic blood pressure (DBP) and antihypertensive usage were recorded preoperatively and postoperatively at 1-year and 2-year. Statistical analysis was performed using SPSSv24.

**Results**

468 (76%) patients were female. The mean age was 46+/-11 years and the mean BMI was 49+/-8 kg/m^2^. 423 (68%) patients underwent LGBYP and the remainder underwent LSG. Postoperatively, the mean BMI fell to 33+/-77 kg/m^2^ at 1-year and stayed the same at 2-year (p<0.001, t-test). The mean SBP decreased from 142+/-19 mmHg preoperatively to 127+/-18 mmHg at 1-year postoperatively, which stayed the same at 2-year (p<0.001, t-test). The mean DBP decreased from 87+/-10 mmHg to 72+/-24 mmHg and 79+/-10mmHg at 1-year and 2-year postoperatively respectively (p<0.001, t-test). 125 (52%) hypertensive patients were off all antihypertensives at 1-year and 2-year postoperatively respectively. 100 (60%) and 94 (57%) patients who were taking 1 or 2 antihypertensives preoperatively were normotensive and off all antihypertensives at 1-year and 2-year postoperatively respectively.

**Discussion**

Improvement in SBP and DBP was sustained over 2 years following bariatric surgery, notwithstanding a 50% reduction in the number of patients on antihypertensives.

### O4 Postoperative atrial fibrillation and long-term risk of stroke and death following cardiac surgery: A systematic review and meta-analysis

#### Muhammad Zain Ali^1^, Peter Chen^2^, Pascal Meyre^3^, Michael Ke Wang^4^, William McIntyre^4,5^, Jeff Healey^4,5^, Richard Whitlock^4,6^, Andre Lamy^4,5^, PJ Devereaux^4,5^, David Conen^4,5^

##### ^1^School of Medicine, Royal College of Surgeons in Ireland, Dublin, Ireland; ^2^University of British Columbia, Vancouver, Canada; ^3^Department of Cardiology and Cardiovascular Research Institute Basel, University Hospital Basel, University of Basel, Basel, Switzerland; ^4^Department of Medicine, McMaster University, Hamilton, Canada; ^5^Population Health Research Institute, McMaster University, Hamilton, Canada; ^6^Department of Surgery, McMaster University, Hamilton, Canada

###### **Correspondence:** Muhammad Zain Ali

**Introduction**

Postoperative atrial fibrillation (POAF) is a frequent complication after cardiac surgery. Studies so far have shown variability in terms of whether POAF is associated with higher risks of stroke or death. To gain additional insights into this important issue, we performed a systematic review and meta-analysis to investigate the long and short-term risks of stroke and death in patients with POAF after cardiac surgery.

**Methods**

We searched PubMed for studies that investigated the association of stroke and death in patients with POAF after cardiac surgery. Studies were included if they reported short (<30 days after cardiac surgery) and long-term risks (>30 days after cardiac surgery) of stroke or death, and had enrolled ≥100 patients. Data were independently abstracted by 2 reviewers and pooled using inverse variance random-effects models. The primary outcome was stroke occurring >30 days after cardiac surgery, and the secondary outcome was all-cause death occurring >30 days after surgery. We presented data as risk ratios (RR).

**Results**

Of the 7131 citations screened, a total of 43 studies met the eligibility criteria. Among the 332,577 participants, 69,205 developed POAF (20.8%). During a mean follow-up of 22 months, patients with POAF had a significantly higher long-term risk of stroke compared to those without POAF (RR, 1.46; 95% confidence interval [CI], 1.29-1.66, I^2^=71%). The risk of stroke was also increased in the first 30 days after surgery (RR for <30 days, 2.63; 95% CI, 2.05-3.38, I^2^=77%). Patients with POAF had an increased long-term risk of death (RR, 1.67; 95% CI, 1.41-1.99, I^2^=90%). There was a trend for increased risk of death during the first 30 days after surgery among patients with POAF (RR for <30 days, 1.73; 95% CI, 0.99-3.02, I^2^=97%).

**Discussion**

Patients with POAF after cardiac surgery have an increased long-term risk of stroke and death.

### O5 Role of SGK-1 mediated ATP production in the survival of extracellular matrix (ECM) detached cells

#### Ishan Antony^1,2^, Jordan Cockfield^1^, Zachary T. Schafer^2^

##### ^1^School of Medicine, Royal College of Surgeons in Ireland, Dublin, Ireland; ^2^Department of Biological Sciences, University of Notre Dame, Notre Dame, Indiana, United States

###### **Correspondence:** Ishan Antony

**Introduction**

Evading both caspase-dependent cell death triggered by extracellular matrix (ECM) detachment (defined as anoikis) and ECM-detachment-induced metabolic defects is necessary for cancer cells to metastasise. We've previously identified a role for SGK-1 downstream of oncogenic Ras to promote survival in ECM-detachment and this study investigates the molecular mechanism by which SGK-1 drives survival in ECM-detached cancer cells with different oncogenic insults.

**Methods**

Cancer cell-lines were cultured in either DMEM media (KPL4, MDA-MB-468, and 4T07 with 1% non-essential amino acids) or McCoy Media (HCT116) with 10%FBS. To examine ATP production, ATP assay kits were used in the parental cell-lines along with over-expression or knockdown of SGK-1. Soft-agar colony formation assays were completed to evaluate anchorage-independent growth. Western blots were run from cell lysates to assess protein abundance and/or phosphorylation.

**Results**

Higher levels of ATP were measured in SGK-1 over-expressed cell-lines. When SGK- 1 was knocked down, significant decreases in ATP were observed in each cell-line. Soft agar plates showed more cell colonies in plates containing SGK-1 over-expressed cells while those with knockdowns of SGK-1 displayed the antithesis. When SGK-1 was over-expressed, we saw an increase in the abundance of GLUT1 protein expression, and when SGK-1 was knocked- down, we saw a decrease in GLUT1 protein expression. Using WZB117 to inhibit the function of GLUT1 decreased the ability of SGK-1 to facilitate glucose uptake.

**Discussion**

In ECM-detachment, we found SGK-1 to be both sufficient and required to promote ATP generation and anchorage-independent growth. Due to the regulation of GLUT-1 expression and its requirement in SGK-1-mediated glucose uptake, SGK-1 plausibly regulates uptake of glucose by coordinating the abundance of this glucose transporter. Future studies would include investigating how SGK-1 regulates the abundance of GLUT1 by examining either transcription factors that promote the transcription of GLUT1 or post-translational factors that regulate the stability of GLUT1 in ECM-detached cancer cells.

## Poster session

### P1 A systematic review of Lutetium-177 PSMA therapy in prostate cancer

#### Amy Pawson^1^, Katherine Zukotynski^2^

##### ^1^School of Medicine, Royal College of Surgeons in Ireland, Dublin, Ireland; ^2^Nuclear Medicine Department, McMaster University, Hamilton, Canada

###### **Correspondence:** Amy Pawson

**Introduction**

Prostate cancer is the second most common cancer and second leading cause of cancer related death in men. Within the last 5-years, a radionuclide agent targeting prostate specific membrane antigen (PSMA) has been assayed for men with metastatic castration resistant prostate cancer (mCRPC). Referred to as ^177^Lu-PSMA therapy, this agent emits targeted radiation. The objective of this review is to assess the effectiveness of ^177^Lu-PSMA therapy in men with mCRPC.

**Methods**

English-language papers evaluating ^177^Lu-PSMA therapy in mCRPC published from January 2012-January 2019 were extracted from Pubmed, Web of Science, Science Direct, and Embase. Excluding those with low patient numbers such as case reports, papers that did not include outcomes and papers where targeted radionuclide therapy (not ^177^Lu-PSMA therapy) were used, yielded 26 papers. Number of patients, dosage schedule, follow-up/outcome measures and side effects were recorded for each study.

**Results**

The number of patients receiving ^177^Lu-RLT and the amount of radioactivity administered, varied across the different studies. Study sizes ranged from 10 to 145 patients, and the dose of treatment ranged from 2 to 9.7 GBq. Studies suggest that the most effective regime is 9 cycles of therapy interspersed by 6 weeks of no treatment. The majority of studies demonstrated a significant response to treatment through a decrease in PSA, as well as a substantial proportion of these studies having a decrease in PSA >50% prolonging median overall survival. Treatment failure was reported in up to 1/3^rd^ of patients, as well as minor side effects.

**Discussion**

Since inception of ^177^Lu-PSMA therapy for mCRPC, several studies have shown effectiveness with increasing overall survival, albeit in the setting of side effects, most commonly xerostomia and thrombocytopenia. More research is needed to establish best clinical practice.

### P2 Adopting the use of a Virtual Clinic to improve the efficiency and standard of care at the Beaumont Hospital General Surgery OPD

#### Avinash Bhupalan

##### Royal College of Surgeons in Ireland, Dublin, Ireland

**Purpose**

This study assessed patients attitudes towards the potential of being seen in a virtual clinic and their current impression of the standard of care and efficiency of the OPD at Beaumont.

**Methods**

Beaumont’s current virtual clinic is being used by the general surgery department to assess a select group of patients post operatively & post endoscopy, in order to assess their general health and scar healing. The end goal is to expand this service to a variety of other departments within the hospital . In order to do this it must first be demonstrated that utilisation of the virtual clinic is more efficient, cost effective and less time consuming for both healthcare providers and patients. Our main objective will be to assess patients’ attitudes regarding the concept of a virtual clinic, whether or not they would be comfortable with this form of care, and whether they believe the same outcome can be achieved in this setting as compared to more traditional settings. To obtain the following information surveys were handed to patients (n=100)in the general surgery outpatient department (OPD) of Beaumont Hospital.

**Results**

From 100 patients surveyed, the average time between leaving one’s house and being seen by a doctor was 1h 44 mins. The average OPD consultation time was 14 minutes. The average waiting in the Virtual clinic (VC) was 4 minutes and 44 seconds, whilst the average consultation time was 5 minutes. The average cost incurred by a patient to get to the Beaumont OPD was €11.55.

**Conclusion**

The virtual clinic is the more cost and time efficient option when seeing a selected group of post operative general surgery patients. The VC saves both hospital and patient a substantial amount of money. In addition to time and cost, it decreases the workload on medical staff.

### P3 Advancing professional healthcare by the use of mentorship

#### Harleen Jhinger^1^, Bridget Murray^2^, Comfort Chima^2^

##### ^1^School of Medicine, Royal College of Surgeons in Ireland, Dublin, Ireland; ^2^School of Nursing & Midwifery, Royal College of Surgeons in Ireland, Dublin, Ireland

###### **Correspondence:** Harleen Jhinger

**Research Questions**

What is the impact of mentorship on the clinical competencies of healthcare professionals?

**Introduction**

Mentorship is a method used to train healthcare professionals across a variety of disciplines. It comprises of a relationship between the ‘mentor’, or the trainer, and a ‘mentee’, or the learner. It is important to determine whether or not mentorship is a useful technique to increase the clinical competencies of healthcare professionals to ascertain where further research should be directed.

**Methods**

A systematic review construct was used to gather data from different sources. Four databases (PubMed, Embase, CINAHL, and the Cochrane Library) were explored using search terms that fell under the categories of “mentorship”, “field of study” and “randomized control trial”, formulating a total of 502 articles, which were applied to an inclusion and exclusion criteria. 82 articles were duplicates, comprising a total of 420 articles through the search strategy. A variety of review methods were employed, including Mendeley, an EBL Appraisal checklist, and the Cochrane Tool of Bias to assess viability of articles, ensure that each article was accounted for once, and minimize the risk of bias.

**Results**

Six articles met the inclusion and exclusion criteria, out of which five displayed mentorship to be a clinically significant interventional method, while one noted no significant difference. From these results, it is noted that mentorship works to improve health outcomes by enhancing healthcare professional clinical competencies.

**Discussion**

Based on this systematic review, mentorship is a strong method to develop clinical competencies of healthcare professionals. In the future, research should be directed towards improving specific mentorship programmes.

**Acknowledgements**

We acknowledge funding received by the Royal College of Surgeons in Ireland Research Summer School.

### P4 Aetiology and functional outcomes following hypoxic ischaemic brain injury

#### Sian Roberts-Walsh^1^, Prasanth Sukumar^1^, Valerie Twomey^2^, Áine Carroll^1,2^

##### ^1^School of Medicine, University College Dublin, Dublin, Ireland; ^2^National Rehabilitation Hospital, Rochestown Avenue, Dublin, Ireland

###### **Correspondence:** Sian Roberts-Walsh

**Introduction**

Hypoxic ischaemic brain injury (HIBI) is a leading cause of long-term neurological disability. The aim of this study was to investigate the aetiology and outcome patients with HIBI admitted to a National Rehabilitation Hospital (NRH) over a 10-year period.

**Methods**

Retrospective analysis of the healthcare records of all patients discharged from the NRH with a ICD 10 code for HIBI from 2000-2018. Descriptive analysis and analysis of the level of disability (using Modified Barthel Index [MBI] and Disability rating scale [DRS]) and discharge destination was carried out using SpSS version 24.0.

**Results**

572 episodes were recorded under the code g93.1. After exclusion of duplicate entries, the number of records analysed was 139. A further 35 were excluded as they did not meet the inclusion criteria. 104 records were reviewed systematically using a standardised proforma.

69 (66%) were male and 35 (33%) were female.

Cardiovascular causes were most common (35.6%), followed by overdose (22.1%). Most had moderate to severe disability on admission (MBI and DRS). Severe disability was associated with respiratory arrest, overdose and neurological causes, while independence was most likely with cardiovascular causes. MBI and DRS improved in the majority (49% and 38.5%) with the greatest improvements seen with cardiovascular aetiology.

45.2 % of patients were discharged to their own home, 18.3% to a nursing home, and 18.3% to an acute hospital.

**Conclusion**

Improvement in functional outcomes following HIBI is correlated to aetiology. This may have implications in helping to predict patient outcome post-HIBI. Study limitations include incomplete recordings on charts and varying sample sizes within aetiological groupings.

### P5 An audit of brain magnetic resonance imagery scans ordered from various departments in multi-speciality hospitals over a 12 week interval

#### Sanat Rashinkar^1^, Sayali Tulpule^2^, Ashish Atre^3^

##### ^1^School of Medicine, Royal College of Surgeons in Ireland, Dublin, Ireland; ^2^Hardikar Hospital, Pune, India; ^3^Chief Neuroradiologist and Paediatric Radiologist, STAR Imaging and Research Centre, Pune, India

###### **Correspondence:** Sanat Rashinkar

**Introduction**

There has been a significant increase in the number of diagnostic imaging requests of the brain from various different departments in the hospitals. It has been observed that a large number of diagnostic imaging requests were related to the brain MRI of the brain causing high workload for the neuro-radiology trainees. This challenge provides an opportunity to evaluate these requests from departments where there is a high demand. The results can improve the radiology department service and reduce workload on the neuro-radiology trainees. This study aims to assess brain MRI scans characterising the departments, indications, and the final diagnosis from the imaging requests for the radiology department.

**Methods**

A retrospective review was conducted using data available from the PACS (Picture archiving and communication system) of a major diagnostic imaging and research centre in Pune. The data was collected on patients who presented to a consultant in a certain department in a hospital in Pune, and patients were asked to undergo a brain MRI scan from this diagnostic imaging centre. The data records for 12 weeks (1/1/2017 to 31/3/2017) were collected which included the age, gender, scan type, department, indications, and final diagnosis of these patients.

**Results**

518 scans were completed. The highest number of requests were from the Neurology and Neurosurgery departments, 62% and 35% respectively. Out of the 518 scans, 36% were MRI Brain Scans, 27% were Brain MRI Angiography, 17% were MRI Brain Epilepsy Control, followed by other types. The most common indication was headache (32.2%) followed by nausea (9.6%) and then vomiting (9.2%). The most common diagnosis was ischemic stroke (11%) followed by haemorrhage (8.1%), demyelinating disease (4.6%), and then others.

**Discussion**

**T**he analysis shows the departments with high imaging requests. The most common indications and final diagnoses were identified. This will assist in improving the radiology department service and aid neuro-radiology trainees.

### P6 An audit to examine why patients were not wearing their identification wristbands

#### David Joyce

##### School of Medicine, Royal College of Surgeons in Ireland, Dublin, Ireland

**Introduction**

The Joint Commission International has highlighted identifying patients correctly as the number one goal to increase patient safety. By ensuring that patients are wearing their identification bands it will decrease the number of issues related to prescribing medicines and having investigations performed. The aim of this audit was to ascertain whether patients were wearing their hospital identification wristbands and whether it was being checked by staff at key risk points. If the patient was not wearing a wristband, the aim was to see who had removed the wristband and why they had removed it.

**Methods**

The audit was undertaken by asking patients whether or not they were wearing their wristbands. The patient was then asked whether they had had their wristbands checked before receiving medications, before an x-ray or scan and before any other investigations (bloods, ECG, clinical exam etc.) If the patient was not wearing a wristband, the patient was asked who removed it and if there was a reason why it had been removed.

**Results**

255 partook in the audit. 95% (n=243) of the patients were wearing wristbands. Most patients (>95%) reported wristbands being checked at various risk points, however, when receiving medication only 76% of patients reported their wristband being checked on the day of the audit. Of the 12 patients who were not wearing wristbands, 50% (n=6) were unsure why it had been removed.

**Discussion**

The results highlighted that most patients were wearing their wristbands and had their identification checked before any procedure. More effort should be made to ensure that all patients are wearing their identification wristbands and to ascertain why some patients had had them removed.

### P7 Analysis of the wild mushroom *Fomitopsis pinicola* for immunostimulatory and anti-inflammatory properties

#### Anum Khalid^1^, Hamna Javed^1^, Chow Lee^2^

##### ^1^School of Medicine, Royal College of Surgeons in Ireland, Dublin, Ireland; ^2^University of Northern British Columbia, Prince George, Canada

###### **Correspondence:** Anum Khalid

**Introduction**

The discovery of new compounds from British Columbia wild mushrooms have shown the ability to either directly inhibit the growth of cancer cells or stimulate the body’s own immune defense mechanism to kill cancer cells. These findings suggest that native fungi may contain potentially powerful natural pharmaceutical agents that should be further studied for medicinal value. In this study, we analyzed six wild mushroom species of *Fomitopsis pinicola* to look for the presence of both immunostimulatory and anti-inflammatory properties that could be of pharmaceutical benefit.

**Methods**

Powdered *Fomitopsis pinicola* was sequentially extracted with 80% ethanol, 50% methanol, water and 5% NaOH. Extracts underwent rotary evaporation and Lyophilization. All samples were used to treat RAW 264.7 mouse macrophage cells after which the supernatant was collected for ELISA testing; Lipopolysaccharide (LPS) and phosphate-buffered saline were used as controls for immunostimulatory and anti-inflammatory testing respectively. Analysis using GraphPad Prism and Kaleidagraph was done. The concentration of sample used to treat the cells was the independent variable while the concentration of TNF-alpha generated as a response was the dependent variable

**Results**

Analysis of TNF-alpha production in *F. pinicola*-treated cells revealed low levels of TNF-alpha as compared to LPS-treated cells. Interestingly, the LPS-induced TNF-alpha production was significantly inhibited by *F. pinicola* when compared to the controls.

**Conclusion**

This study showed that the wild mushroom species *Fomitopsis pinicola* possess notable anti-inflammatory properties and low immunostimulatory activity. Possible influencing factors include unknown systematic error, sample contamination, and human error. The relevance of this study is that it implicates promising biological anti-inflammatory activity in BC wild mushrooms that can be of pharmaceutical benefit.

### P8 Analyzing characteristics of Hawaii patients with mixed seizures

#### Katharine Cater^1,2^

##### ^1^Hawaii Pacific Neuroscience, Honolulu, Hawaii, United States; ^2^Tripler Army Medical Center, Honolulu, Hawaii, United States

**Objectives**

To analyze characteristics of Hawaii patients with a dual diagnosis of Psychogenic Non-Epileptic Seizures (PNES) and Epileptic Seizures (ES) with the aim of gaining a better understanding of the patterns seen in patients with PNES and ES in Hawaii.

**Methods**

VEEG results were used to select patients with both a conversion disorder with seizures and an epilepsy diagnosis. Patient characteristics, epilepsy type, and psychological comorbidities were then analyzed to find patterns. These patterns were then compared with patterns of patients with the same dual diagnosis in other populations

**Results**

Nine patients were found, with an average age of 53.6. They are on average borderline obese, mostly female, and Asians and Pacific Islanders. Focal (not intractable) epilepsy was the most common epilepsy type, and there were 10 reported psychological comorbidities across 9 patients. None of the patients had a family history of epilepsy.

**Conclusions**

In patients in Hawaii with both ES and PNES, the average age is older than seen in previous studies. It was most prevalent in Asians and Pacific Islanders. Other findings such as predominate sex, the type of psychological comorbidities, and the type of coexisting epilepsy were common to other studies.

### P9 Assessing parental concerns of the recovery process of pediatric burn patients

#### Youlim Song^1^, Katerina Green^2^, Aoife Reilly^2^, Emily Hernandez^3^, Skylar Freyman^1^, Abigail Fenton^4^, Susan Zeigfeld^5^, Dylan Steward^5^, Carisa Parrish^5^

##### ^1^Johns Hopkins University, Baltimore, United States of America; ^2^Royal College of Surgeons in Ireland, Dublin, Ireland; ^3^California State University Northridge, Los Angeles, United States of America; ^4^Our Lady of Good Counsel High School, Olney, Maryland, United States of America; ^5^Johns Hopkins Medical Institution, Baltimore, United States of America

###### **Correspondence:** Aoife Reilly

**Introduction**

Parents of pediatric burn patients are at significant risk of psychological distress, with up to 50% of parents reporting clinically elevated levels of Post Traumatic Stress Disorder symptoms. Clinical experience suggests that a significant source of parental distress is the visible nature of the injury. Perception of skin appearance affects the quality of life in pediatric patients with other visible skin conditions. Research is limited on how skin tone and injury affect psychosocial adjustment in pediatric burn patients. The aim of this study was to investigate the effect of pediatric burn cosmetic outcomes on parental concern to assess if educational pictures of other children’s burns healing over time could address these concerns.

**Methods**

An anonymous self-report survey was administered to parents (n=61) assessing their concerns about their child’s adjustment to burn and skin healing, as well as their interest in additional education about the skin healing process. Data collection is ongoing.

**Results**

Skin appearance (n=29; 47.5%) was the most frequently endorsed parental concern, followed by Child Pain (n=28; 46.9%). In total, 42 parents (69.0%) reported that educational pictures showing how other children’s burns healed over time would help manage their expectations of how their child might heal. The majority of parents (n=43; 70.5%) endorsed that access to photographs of wound and burn scar healing process of other pediatric burn patients with similar skin tones would help them understand their child’s recovery course.

**Discussion**

Education regarding cosmetic outcomes among pediatric burn patients could be a beneficial tool in addressing parental concerns about the healing process. Further –scale studies are required to validate these findings.

### P10 Association of sub-clinical and clinical hypothyroidism with non-alcoholic fatty liver disease: A retrospective hospital based case control study

#### Radhe Shah^1^, Shrutav Patel^1^, Pooja Rangwala^2^, Ayush Thakkar^3^, Rudra Patel^1^, Anita Verma^1^

##### ^1^Smt. NHL Municipal Medical College, Ahmedabad, India; ^2^AMC MET Medical College, Ahmedabad, India; ^3^GCS Medical College, Hospital and Research Centre, Ahmedabad, India

###### **Correspondence:** Radhe Shah

**Introduction**

Non-Alcoholic Fatty Liver Disease (NAFLD) is a well-established risk factor for various hepatic and extrahepatic morbidities. Hypothyroidism tends to affect lipid and carbohydrate metabolism, a pathology also seen in patients with NAFLD. However, the role of hypothyroidism in causing NAFLD is not yet well defined. Therefore the aim of the present study was to find an association between NAFLD and hypothyroidism.

**Methodology**

The cross-sectional study was conducted among 250 Indian subjects visiting SVP Hospital between July-September 2019. The population consisted of 100 cases defined by Ultrasonography (USG) confirmed findings for NAFLD and 150 controls with USG findings negative for NAFLD; matched for age, sex, BMI and without history of alcohol use. Personal history of hypothyroidism and altered thyroid function tests were used to classify clinical (defined by TSH(>5.5uIU/mL) and FT4(<0.8ng/dl), FT3(<2.1ng/dl), T4-SERUM(<3.2ug/dl), T3- SERUM(<0.63ng/ml)) and subclinical hypothyroidism(defined by TSH(>5.5uIU/ml) and FT4, FT3, T4-serum and T3-serum: normal). Analysis of the data was carried out using Odds ratio and Chi Square Test.

**Results**

The mean age and BMI of the total subjects were 50.96±17.34 and 24.55±5.74 respectively, 53% of which were females. Out of the total cases, 23% and 17% had clinical and subclinical hypothyroidism respectively. A greater occurrence of clinical and subclinical hypothyroidism was found in cases as compared to the controls with the Odds Ratio being 1.41 and 1.23 respectively. However, Chi Square showed non-significant results for association of Clinical hypothyroidism with NAFLD(p=0.2832) and Subclinical Hypothyroidism WITH NAFLD(p=0.561). Among the cases with hypothyroidism, 60% were prehypertensive and 27.5% were hypertensive; 75% were diabetics.

**Discussion**

No significant association was found between the spectrum of hypothyroidism and NAFLD in this study. Multifactorial causation of NAFLD, along with restriction of the study population to hospital patients are the major lacunae of this study. Further research is recommended to confirm the results.

### P11 Asymptomatic carotid disease - Time for some consensus

#### Husam Alhuwaish^1^, Peter Naughton^1,2^

##### ^1^School of Medicine, Royal College of Surgeons in Ireland, Dublin, Ireland; ^2^Beaumont Hospital, Dublin, Ireland

###### **Correspondence:** Husam Alhuwaish

**Introduction**

Management of asymptomatic carotid stenosis is controversial. Even though the majority of practice guidelines provide Class-I evidence supporting carotid endarterectomy (CEA) in >60% asymptomatic carotid stenosis (ACS), many experts disagree with surgery in ACS.

**Objective**

To perform a review of randomized controlled trials (RCTs) looking at carotid surgery versus no carotid surgery for asymptomatic carotid stenosis (ACS) and to compare evidence with current international guidelines.

**Methods**

MEDLINE (pubmed) search was conducted looking for RCTs published in the time period 1980-2018 comparing medical therapy alone to surgery in the management of ACS. Also a review of practice guidelines was done.

**Results and Conclusions**

Guidelines are based on major RCTs that were published in the 1990s such as VA trial -1993, ACAS trial -1995, and ACST trial - 2004. All compared aspirin to CEA.1990s best medical therapy (BMT): Aspirin. ‘Modern’ BMT: intensive use of a wide range of anti-platelets, newer antihypertensives, potent statins, diabetes control, Mediterranean diet, and smoking cessation. ‘Modern’ BMT is superior to aspirin and might supersede surgery. The 10-year ARR is only 4.6% in ACST trial making around 95% of CEA unnecessary and this results overtreatment that is not cost-efficient. Because BMT is improving and the NNT is increasing, many seem to disagree with guidelines. No published RCT was found comparing ‘modern’ BMT to surgery in asymptomatic carotid stenosis patients. CREST-2 has two parallel studies comparing ‘modern’ BMT alone vs. CEA plus BMT and BMT alone vs. CAS plus BMT in 2480 participants; CREST-2 will be published in 2020.

### P12 Capacity of melatonin against hyperglycemia induced ischemia in heart tissue of alloxan diabetic rats

#### Kyrylo Pantsiuk^1^, Oleksandra Kushnir^1^, Nataliya Pantsyuk^2^, Andriy Pantsyuk^2^

##### ^1^Department of Bioorganic Chemistry and Clinical Biochemistry, Higher State Educational Establishment of Ukraine, Bukovinian State Medical University, Chernivtsi, Ukraine; ^2^Higher State Educational Establishment of Ukraine, Bukovinian State Medical University, Chernivtsi, Ukraine

###### **Correspondence:** Kyrylo Pantsiuk

Since the heart is the target organ for coronary atherosclerosis and ischemia, we have studied the role of melatonin against alloxan-induced heart toxicity in rats.

The aim was to determine the influence of melatonin on basal levels of glucose (BG) and HbA1c, activity of pyruvate kinase (PK) [EC 2.7.1.40] in the heart of alloxan diabetic rats under conditions of melatonin injections.

The experiments were carried out on 60 sexually mature male albino rats with the body mass – (0,18 – 0,20) kg. Alloxan diabetes was evoked via injecting the rats with a 5% solution of alloxan monohydrate intraperitoneally in a dose of 170 mg/kg of body weight. The animals were divided into groups: 1) control; 2) diabetic; 3) diabetic animals which were introduced the melatonin preparation intraperitoneally in a dose of 10 mg/kg of b.w. at 8 a. m. daily during 42 days starting with a 5-th 24 hour period after the injection of alloxan. Statistical analysis of results was conducted by Student’s test. Sufficient level considered probability differences p≤ 0,05.

We got an increase of BG as well as HbA1c on 120 and 80% compared with control. Hyperglycemia caused degenerative changes in heart tissue including ischemia formation through development of microangiopathy. Same time the activity of PK was found to be decreased on 42% in comparison with control. It can be explained by low uptake of glucose from the blood to heart muscle in conditions of insulin deficiency that leads to a reduced rate of glucose metabolism. Melatonin led to normalization of BG level and markedly decreased of HbA1c. This was conducted with increase the activity of PK to normal value.

It can be concluded that the administration of melatonin notably recovered the heart from hyperglycemia induced antioxidant imbalance, inflammation and apoptosis as well as rectified the imbalance in carbohydrate metabolism.

### P13 Carriage of potential pathogens in hands of healthcare workers

#### Uditha Udumulla, Anjalee Thilakasiri, Aara Thilfar, Dilumi Thirasarie, Sandun Thiwanka, Shermila Ubhayaratna, Shalika Ediriweera, Muditha Vinolaka, Ashan Waduge, Hansini Waidyarathne, Asela Ekanayake, Nilanthi Dissanayake

##### Department of Microbiology, Faculty of Medicine, University of Peradeniya, Peradeniya, Sri Lanka

###### **Correspondence:** Uditha Udumulla

Pathogens carried by the hands of health care workers contribute to a significant proportion of HAIs. This study was conducted to evaluate the pathogenic organisms carried by hands of health care workers with the objectives of identification of pathogenic organisms carried by the hands of health care workers with the antibiotic sensitivities and to assess the differences in hand carriage of pathogenic organisms according to staff category, unit and the hand hygiene performance. This study was conducted at Teaching hospital, Peradeniya.

Total sample size needed was calculated and identified the minimum numbers of hand imprints needed in each staff category by using stratification method. Accordingly, 117 Hand imprints of the dominant hand were collected from 117 HCWs onto blood agar plates just before attending to a patient. Plates were incubated overnight at 35^o^C and organisms identified according to standard microbiological techniques. Antibiotic sensitivity performed according to CLSI methodology. Data analyzed with SPSS version 20.0.

117 hand imprints were collected from 18 doctors, 52 nurses and 47 health care assistants. 44 pathogens were grown from 42 (35.89%) of the samples. Commonest organism isolated was *Acinetobacter* spp. and it accounted for 18.8% of hand carriage. Coliforms carried by 11.96% , *S.aureus* by 5.13% and *Pseudomonas* spp. by 1.71%. 11 MDR organisms were isolated accounting for 9.4% of hand carriage. No association was found among pathogen carriage/MDR organisms and the staff category, unit or performance of hand hygiene.

A considerable proportion of health care workers were carrying pathogenic organisms in their hands. Most of them were Gram negatives of which some were MDR organisms.

**Keywords:** Hand carriage, health care workers, hand imprints, MDR organisms

### P14 Can novel cancer driver genes be identified by analysing mutations in cancer genomes? Using whole genome sequencing data of patients with colorectal cancer to identify the mutation patterns

#### Xiangmei Cui, Simon Furney, Valentina Thomas, Razi Alalqam

##### School of Medicine, Royal College of Surgeons in Ireland, Dublin, Ireland

###### **Correspondence:** Xiangmei Cui

Colorectal cancer is a leading cause of mortality and morbidity in modern society. Colorectal cancer is associated with specific gene mutations. This research aims to analyse the genome using next generation sequencing techniques. The study evaluated patients with colorectal cancer in China. Next generation sequencing, with low costs, has been granted by health practitioners worldwide with its unprecedented level of accessibility to vast genomic information regarding their patients. In this study, the R open statistical computing software has been used, while referring to COSMIC (Catalogue of Somatic Mutations In Cancer) to interpret global colorectal cancer genomic data provided by the ICGC (International Cancer Genome Consortium) Data Portal.

This research is a stand-alone project and the objective was to de-duplicate irrelevant data and consequently convert the patient mutation data into 31 different signatures. The percentage of these specific signatures would then allow us to deduce potential aetiologies for the genome mutations from tumour samples, from anonymised clinical data. In this study, we analysed 325 patients, with more than 6,000,000 mutation data points, for patients with colorectal carcinoma from China, using the R open statical computing software. The information was then deconstructed and different graphs were produced for all unique sample ID’s, allowing for the visualization of weights of different mutation patterns. An additional command was used to identify the samples that are microsatellite instable, which is also known as Lynch syndrome, which is defect in the mismatch repair genes MSH2, MLH1, PMS1 and PMS2.

In conclusion, 259 of 325 patients’ genome mutation can be classified as microsatellite instable. This means many patients with colorectal cancer inherited the genes from their family. In addition, Signature 1, 5, 6, 10 were the most common mutational signatures in this data, which is similar to the COSMIC data provided.

### P15 Characteristics of a novel community based step-down intermediate care unit – The St. Francis Unit, Cork City, Ireland

#### Kwun Hang Aaron Ho^1^, Henry Smithson^2^

##### ^1^School of Medicine, University College Cork, Cork, Ireland; ^2^Department of General Practice, University College Cork, Cork, Ireland

###### **Correspondence:** Kwun Hang Aaron Ho

**Introduction**

St. Francis Unit (SFU) is a community based step-down intermediate care unit of the Mercy University Hospital (MUH) in Cork, which serves to allow transitional time for medically stable patients to regain functional independence prior to discharging home.

**Aim**

We aim to evaluate the patient characteristics of SFU and determine the patient demographics, unit-admission reasons, length of stay (LoS) and place of discharge.

**Methods**

A prospective cohort study was performed on the first 314 consecutive patients admitted to SFU between September 2016 to May 2017. Patients were included if they were admitted to MUH, no longer required acute inpatient care, and unfit for home discharge. Data were collected by the head-nurse, validated through weekly multi-disciplinary-team meetings and analysed using SPSS with p<0.05 considered significant.

**Results**

Of 314 patients, 173 (55%) were females and mean age was 79+/-9 years. 108 (34%) patients were 70-79 years-old, 145 (46%) were 80-89 years-old and 25 (7%) were ≥90 years-old. Resolving infection was the commonest reason for unit-admission (43%), compared to gaining mobility (21%), postoperative recovery (13%), neurological disorders (11%), circulatory disorders (11%), gastrointestinal disorders (3%), respiratory disorders (2%) and soft-tissue infections (2%). The mean LoS was 11+/-7 days: <7days (29%), 8-14days (41%), 15-21days (14%), 22-28days (2%) and >28days (3%). For place of discharge, 213 (68%) patients were discharged home, 35 (11%) were readmitted to MUH and 21 (7%) were transferred to long-term care. Age was positively correlated to infection (OR 1.04; 95%CI 1.01 to 1.06; p<0.013) but not LoS (OR -0.13; 95% CI -0.17 to -0.14; p=0.83).

**Discussion**

SFU patients were mostly elderly females between 80-89 years-old with mean LoS of 11 days, with resolving infection as the commonest reason for unit-admission. There was no significant correlation between LoS and reason for unit-admission, which suggest possible underlying psychosocial factors in influencing LoS.

### P16 Cholesterol-modification of an Ebola peptide fusion inhibitor to increase its antiviral potency

#### Talal Almas, Marc Devocelle

##### Royal College of Surgeons in Ireland, Dublin, Ireland

###### **Correspondence:** Talal Almas

**Introduction**

Ebola and Marburg are *Filoviruses* that cause severe haemorrhagic fever in humans. Ebola and Marburg require fusion to a host cell- membrane, which is facilitated by the enveloped Glycoprotein 2 (GP2). GP2 possesses similarities to the HIV GP41 peptide, inhibitors for which have been described, but synthesis of such fusion-inhibitors for *Filoviridae* remains challenging.

**Methods**

A 40-mer viral peptide was synthesised using the CEM Liberty Blue peptide-synthesiser. A 2-Chlorotrityl Hydrazine resin was used as solid-support to modify the C-terminus with a hydrazide group, enabling us to conjugate the peptide to the oxidized 4-Cholestene-3- one. To test the resin, a model 12-mer sequence was synthesised. Chromatographic analysis was carried out by RP-HPLC using Gemini 5 μm C18 110 Å columns. The mobile-phase consisted of buffer A: 0.1% TFA in water and buffer B: 0.1% TFA in acetonitrile, with a gradient of over 30 min at a flow rate of 1 ml min−1. Mass spectrometry (MS) was performed by ESI MS and recorded on Advion Expression.

**Results**

Attempts have been made to produce a peptide conjugated via a thioester bond to the side-chain of a cysteine residue added to the C-terminus, but have failed to yield any activity against Ebola and Marburg. We suspect that this could be, in part, due to the unwanted Cysteine interactions associated with a non-cyclical peptide, and due to the unstable thioester bond. We therefore obtained the correct mass spectrometry peaks for the described peptide sequence by circumventing these issues.

**Discussion**

We expect that modification of the fusion entry-inhibitor peptide with 4-Cholestene-3-one will increase its antiviral potency. Although lipidated antiviral peptides for HIV have been described, they cannot be extrapolated to viruses such as Ebola; our novel synthetic approach therefore aims to target Ebola and Marbug viruses, and to define a broader scope for antiviral-peptide application.

### P17 Comparison of percutaneous endoscopic, open and laparoscopic gastrostomy tube placement techniques in children: A systematic review and meta-analysis

#### Nasser Monzer^1^, Axel Ivander^1^, Ahmad Zaghal^2^

##### ^1^Royal College of Surgeons in Ireland, Dublin, Ireland; ^2^Department of Surgery, American University of Beirut Medical Centre, Beirut, Lebanon

###### **Correspondence:** Nasser Monzer

**Introduction**

Gastrostomy tubes (G tubes) are frequently placed to provide feeding routes in paediatric patients. They can be inserted via a number of techniques; this study focuses primarily on the percutaneous endoscopic (PEG), laparoscopic (LAP) and open surgical (OPEN) techniques. The aim of this study is to review and compare the clinical outcomes and major complication rates in these insertion techniques.

**Methods**

A systematic review of published comparative articles in the last 20 years was conducted on the following databases: PubMed, MEDLINE, Cochrane and EMBASE. Studies that included any concomitant procedures such as the Nissen Fundoplication were excluded from the study. Two reviewers independently carried out the data extraction and data analysis stages. Primary outcomes included major complication rates, operative times, and demographic associations.

**Results**

After screening databases, 11 studies including a total of 1,925 patients met the inclusion criteria. Patients in the group undergoing LAP procedures had a significantly lower risk of developing major complications when compared to patients in the PEG and OPEN groups (PEG vs LAP: OR=3.04; 95% CI=1.42-6.51; P=0.0043) (LAP vs OPEN: OR=0.22; 95% CI=0.05- 0.95; P=0.043). There was no significant difference in major complication rates between the PEG and OPEN groups. The most frequently reported major complications were the formation of gastro-cutaneous and gastro-colonic fistulas.

**Conclusions**

This review suggests that the insertion of G tubes without any concurrent procedures is safest when using the LAP approach. Studies with better quality, such as randomised controlled trials, are needed in order to provide a more definitive answer as to which approach holistically yields the greatest results.

### P18 Comparison of Apple Watch Series 4 and KardiaMobile: A study of popular consumer-operated heart rate trackers (eHeaRT)

#### Charles Lee^1^, Mr. Calvin Lee^1^, Dr. Chi-Ming Chow^2^

##### ^1^School of Medicine, Royal College of Surgeons in Ireland, Dublin, Ireland; ^2^St. Michael’s Hospital, Toronto, Canada

###### **Correspondence:** Charles Lee

**Introduction**

Products such as the Apple Watch and KardiaMobile have become widely available for heart rate and rhythm monitoring. However, little is known about their application in day-to-day clinical activities and the accuracy. In this study, we sought to evaluate the ability for the Apple Watch Series 4 and KardiaMobile to correctly diagnose heart rhythm and rate.

**Methods**

127 patients among the Toronto Heart Centre (THC) and CareFirst were recruited in the summer of 2019. Each patient had a 12-lead ECG recorded and ECG recordings taken from the Apple Watch Series 4 and KardiaMobile.

**Results**

Among the patients, 81.89% had sinus rhythm and 18.11% had atrial fibrillation. Of the 107 heart rhythm readings each from KardiaMobile (KM) & Apple Watch (AW), KM readings were 90% correct while 1% incorrect and 9% inconclusive; AW on the other hand had 83% correct with 1% incorrect and 16% inconclusive. Moreover, the KM readings had an accuracy of 98.96% whist AW was 98.88%, with slight variations accounting for random error. However, KM heart rate readings had 69% correct & 31% incorrect whilst AW ECG readings had 75% correct & 25% incorrect; AW light readings had 68% correct & 32% incorrect.

**Discussion**

The findings suggest that KM & AW both accurately detected heart rate and heart rhythms. Moreover, the AW ECG heart rate readings were more accurate than KM. However, both products have inherent limitations in practical settings. The AW ECG requires users to hold their finger on a small button at the side of the watch, which was difficult for the elderly, many of whom experienced tremors which interfered with the readings. Whereas, KM required distancing of other electronics due to their interference with KM ECG recordings. AW is also about 5-6 times more expensive than KM which limits its availability to our patients.

### P19 Correlation of myofascial trigger points with shoulder pain and function in post-stroke patients with painful shoulder

#### Mahnoor Asif, Yasha Sajjad, Fizza Nasir, Saima Zahid, Danish Hassan

##### Riphah International University, Lahore, Pakistan

###### **Correspondence:** Mahnoor Asif

**Introduction**

Myofascial trigger point (MTrP) is a localized hyperirritable spot in the taut band of muscles. Palpation is the only means by which myofascial trigger point is diagnosed. Hemiplegic shoulder pain can be a chief issue in stroke patients. Myofascial trigger points can be the source of this pain and magnify the shoulder disability in this population. The aim of this study was to find the correlation of MTrPs with shoulder pain and function in stroke patients with painful shoulder.

**Methods**

It was a cross sectional study and data was collected from March 2019 to June 2019. Non-probability convenient sampling was used. A sample size of 70 patients was taken. Data was collected from Pakistan Society for the Rehabilitation of the Disabled (PSRD) Lahore, Mansoorah Hospital Lahore, Jinnah Hospital Lahore, Ittefaq Hospital Lahore, Riphah Rehabilitation Clinic Lahore, Punjab Institute of Neuroscience (PINS) Lahore General Hospital, and Lahore Occupational Therapy Welfare Centre. Post-stroke patients with shoulder pain were the target population. Level of pain was recorded by Numeric Pain Rating Scale (NPRS). Shoulder disability was assessed by The Disabilities of the Arm, Shoulder and Hand (DASH) Score. Data was analyzed by using Statistical Package for Social Sciences (SPSS) 21. Spearman Correlation test was used for correlation.

**Results**

MTrPs are moderately correlated with shoulder disability for supraspinatus, infraspinatus and upper trapezius (r= 0.52, r= 0.54 and r= 0.50 respectively). MTrPs are moderately correlated with shoulder pain for upper trapezius, infraspinatus and supraspinatus (r= 0.50, r= 0.52 and r= 0.50 respectively).

**Discussions**

This study found that myofascial trigger points are moderately correlated with shoulder pain and shoulder disability in post stroke patients. Because of this correlation, trigger points should be specifically examined and treated for better prognosis in population with hemiplegic shoulder pain.

### P20 Delineating the retrosplenial portion of cingulum bundle: A quantitative methodological comparison of three common tract delineation techniques

#### Ahmed Zainy^1^, David Coppinger^1^, Elena Roman^2^, Alannah Grealy^2^, Erik O'Hanlon^2^, Mary Cannon^3^

##### ^1^Royal College of Surgeons in Ireland, Dublin, Ireland; ^2^Trinity College Institute of Neuroscience, Dublin, Ireland; ^3^Department of Psychiatry, Royal College of Surgeons in Ireland, Dublin, Ireland

###### **Correspondence:** Ahmed Zainy

**Introduction**

The cingulum is a key bundle in the limbic system. Diffusion Imaging is a technique utilised to study the integrity and the density of white matter. There is inconsistency in diffusion imaging studies with some studies identifying abnormal white matter in psychotic disorder while others fail to find differences. This inconsistency in findings can partly be attributed to the fact that different methods have been used across studies. Hence, this research will discuss three different methods applied to the same diffusion data to analyse, extract and investigate the cingulum.

**Methods**

Data from a case-control sample of 11-13 years adolescents (25 with psychotic experiences and 25 age and gender matched controls) was acquired. MRI High Angular Resolution Diffusion-Imaging (HARDI) featuring Constrained Spherical deconvolution (CSD) based fibre tractography, was used. Following the Jones et al 2013 protocol, the cingulum was divided into four sections (retrosplenial, body, posterior, and hippocampal portions). Three tract delineating methods were employed. Subject-specific anatomically based (Gold standard), semi-automated “atlas-based”, and Standard Atlas Template based tractography. Comparisons were performed in SPSS24 to FA, MD, AD and RD metrics for the bilateral retrosplenial portions.

**Results**

Significant differences were identified between Standard Atlas Template measures when compared to those from Gold standard and semi-automated methods, FA p<=4.4E-64, RHS MD p<=7.2E-7, RHS AD p<=1.6E-12 and RD p<= 8.6 E-59. Nevertheless, “Gold standard” and semi-automated diffusion metric measures were very similar (p<=7.0 E-7 in the retrosplenial portion).

**Conclusions**

These findings outline the importance of cross study comparisons when tract methodologies are different. The Standard Atlas approach while useful as a general comparative white matter method localised to the cingulum, is not directly comparable to the more anatomically accurate subject-specific and semi-automated methods. In contrast, the semi-automated results were accurate enough to suggest using it as a more time efficient, yet reliable method.

### P21 Describing fertility in men with cystic fibrosis

#### Christine O’Keefe^1^, Katherine Griffin^2^, Jenna Sykes^2^, Diana Elizabeth Tullis^2^

##### ^1^School of Medicine, Royal College of Surgeons in Ireland, Dublin, Ireland; ^2^Department of Respirology St. Michael’s Hospital, Toronto, Canada

###### **Correspondence:** Christine O’Keefe

**Background**

Infertility in males with Cystic Fibrosis (CF) is well documented, with around 98% of men demonstrating azoospermia secondary to congenital bilateral agenesis of the vas deferens (CBAVD). As the structures for spermatogenesis (i.e. the testes and caput epididymis) remain intact, there is the potential for men with CF to father biological children through assisted reproductive technology, with pregnancy rates varying from 30-60%. Literature in the area of fatherhood in men with cystic fibrosis is scarce, and to date, there have not been any large scale studies conducted. The aim of this study, therefore, is to better understand the knowledge, attitudes, and practices of men with CF regarding their reproductive potential and options for fertility treatment.

**Methods**

A survey will be distributed to males aged 20-75 years currently in attendance at the adult CF clinic at St. Michaels Hospital during their routine clinical visit. The survey was created following a review of pertinent literature and interviews with CF patients and physicians. Questions were grouped into three sections: demographic information, knowledge and attitudes regarding fertility in men with CF, and experiences in starting a family. Out of pocket costs are included as a potential point of comparison with other CF populations worldwide. Descriptive statistics will be used to analyse responses.

**Expected Results**

There are 324 males currently in attendance at the clinic. The survey is 59 questions in length; as participants will only answer a subset based on their current situation, they will be divided into one of four groups for analysis. It is expected to take 1 year to collect the required data.

**Discussion**

By understanding how men with CF view their fertility and what assistance they may require from their health care provider, there is the potential for improving quality of care and uncovering new areas of research.

### P22 Determining if the methods put in place to reduce unnecessary free thyroid testing at Michael Garron Hospital have been successful

#### Elena Colussi-Pelaez^1^, Rebecca Fine^2^, Raymond Fung^2^, Zoe Lysy^2^

##### ^1^School of Medicine, The Royal College of Surgeons, Dublin, Ireland; ^2^Michael Garron Hospital, Toronto, Canada

###### **Correspondence:** Elena Colussi-Pelaez

**Introduction**

TSH is recommended as the screening tool for hypo/hyperthyroidism. In most cases, TSH is sufficient for screening of disease. Often, free T4 and free T3 are also ordered by clinicians, even when TSH is subsequently normal: this has been openly discouraged by Choosing Wisely Canada, Endocrine Section^3^ and labelled “unnecessary test”. These tests often increase healthcare costs without any added clinical benefit. There is also little value to duplicating thyroid testing within 2-4 weeks given time to equilibrium. At Michael Garron Hospital (MGH), we decided to put in place measures to reduce unnecessary thyroid testing via automatic prompts in the computer ordering system Power Chart. This study was conducted to determine if the methods put in place to reduce unnecessary free thyroid testing at MGH have been successful.

**Methods**

In fall 2018, changes to the ordering system were made. We then collected all thyroid testing data at MGH which was collected over a 3-month period (from January-March 2019) to review ordering patterns and compare with data prior to interview (2018 data was collected previously). All thyroid testing was divided into 4 following mutually exclusive groups. The charts of the patients were reviewed and the tests (TSH, Free T4, Free T3) were identified as normal/high/low. The duplicate testing was also be reviewed i.e. TSH tests ordered within 1 month of each other and FT3/FT4 tests ordered within 2 weeks of each other were considered duplicate.

**Results**

The majority, 78% of total TSH ordered were ordered alone, 98% being showed a normal result. Moreover, 27% of TSH ordered with FT3/FT4/both were normal.

**Discussion**

To conclude, the methods used to reduce unnecessary free thyroid testing at MGH have proven successful as there was a 9% decrease in the amount of additional free thyroid testing ordered over a 3-month period compared to last year.

### P23 Development of a pre-clinical test to detect dysfunctional haemostasis

#### Aleece Warner^1^, Aisling Rehill^2^, Roger Preston^2^

##### ^1^School of Medicine, Royal College of Surgeons Ireland, Dublin, Ireland; ^2^Department of Molecular and Cellular Therapeutics, Royal College of Surgeons Ireland, Dublin, Ireland

###### **Correspondence:** Aleece Warner

**Introduction**

Haemostasis is the natural process of maintaining constant blood flow through vessels. Inappropriate thrombin or plasmin generation can result in dysfunctional haemostasis leading to hypercoagulable or haemorrhagic disorders. This study aimed to generate and compare haemostatic profiles for a cohort of healthy plasma samples and assess coagulation and fibrinolysis.

**Methods**

The thrombin generation assay (TGA) protocol was used to assess the coagulation cascade and the effect of tissue factor (TF) on haemostatic parameters. A novel developed plasmin generation assay (PGA) was used to evaluate the fibrinolytic pathway and the effect of thrombin and thrombomodulin (TM) on fibrinolysis. Data was analysed using Microsoft Excel and GraphPad Prism.

**Results**

Plasma containing high TF generated a higher peak thrombin, higher endogenous thrombin potential and a shorter lagtime compared to plasma with low TF. Plasmin generation, mediated by tissue plasminogen activator (tPA), was increased by the presence of thrombin, but inhibited by the presence of TM. TGA and PGA parameters were not impacted by sex, blood groups or age.

**Discussion**

TF is essential for initiation of the coagulation cascade as higher levels resulted in shorter lagtime, higher peak thrombin and thrombin generation. Thrombin in complex with fibrin increases fibrinolysis through the stimulation of tPA activity. TM in complex with thrombin inhibits fibrinolysis via the activation of thrombin activatable fibrinolysis inhibitor (TAFI). This research is relevant as it provides baseline haemostatic parameters for comparison to diseased cohorts. It will aid in identifying individual parameters that determine bleeding phenotype and subsequently help predict bleeding risk in patients with bleeding disorders.

### P24 Development of an opiate mortality review board in Erie County, New York, USA

#### Campbell Shaw^1^, Laurene Tumiel-Berhalter^2^, Caroline Horrigan^2^

##### ^1^Royal College of Surgeons in Ireland, Dublin, Ireland; ^2^Department of Family Medicine, University of Buffalo, Buffalo, United States of America

###### **Correspondence:** Campbell Shaw

**Introduction**

Opiate mortality is on the rise in Erie County, New York and mortality levels have tripled between 2012-2016^1^. The introduction of prescription drug monitoring in New York State has led to an increase in individuals abusing illegally obtained opiates, which are often more toxic than those prescribed by health care practitioners. Development of an Opiate Mortality Review Board is underway to investigate the circumstances that lead to addiction in these individuals and using the information acquired to mitigate future deaths following drug abuse.

**Methods**

To develop an OMRB, the team created a questionnaire for interviewing close- contacts of opiate mortality victims. This required analyzing current successful OMRBs in other countries. The template was also developed based on the national Fetal Infant Mortality Review Board, posing a similar format. Sections in the questionnaire included: a summary, consent forms, FAQ’s and the interview guide. The guide sections included: demographics, trauma, education, employment, family, healthcare utilization and past medical history.

**Results**

The OMRB involves collecting data and executing a plan. Mortality cases following overdose were selected at random. The Case review team analyzed the autopsy report and reached out to close contacts of the deceased for further insight on their lifestyle. Information gathered by the case review team was used to implement a plan to be carried out by a community action team. The CAT involves community members and offered addiction aid services, often in underserved areas of Erie County where abuse is more prevalent.

**Discussion**

The methods of collection and personnel have been established to execute the mission. As opiate abuse has become a national epidemic, a successful OMRB in New York State will be a template for development of review boards in other states suffering from this epidemic.

### P25 Dexamethasone efficacy for acute pain management after hip arthroplasty

#### Natalija Buraka^1^, Iveta Golubovska^1^, Aleksejs Miscuks^1^, Lauris Repsa^2^, Sergejs Zadoroznijs^1^

##### ^1^University of Latvia, Riga, Latvia; ^2^The Hospital of Orthopaedics and Traumatology, Riga, Latvia

###### **Correspondence:** Natalija Buraka

**Introduction**

Dexamethasone is used to control inflammation, edema, but also it has a great analgesic effect. By enhancing endogenous opioid synthesis there is potential to reduce high doses of narcotics . Hyperglycemia is a side effect of dexamethasone. Lactate may be raised too. It affects the dynamic of treatment. The aim was to investigate dexamethasone’s effect on pain, serum glucose and lactate for patients after hip replacement.

**Methods**

This prospective randomized study includes 50 patients undergoing hip arthroplasty. Surgery was performed under spinal anesthesia. Multimodal analgesia was administered for both groups. The experimental group additionally received dexamethasone 8 mg i/v before surgery and 4 mg i/v 6 and 12 hours after the first dose. Rescue analgesic – morphine 10 mg s/c was administered if needed. Glucose and lactate were measured before surgery, at 18:00 and 6:00 the next day. Pain level was measured by VAS for the perioperative period. Data were processed using SPSS program.

**Results**

Pain level during rest in the experimental and control group accordingly was - 1.2 and 3.1 at 18:00; 0.8 and 2.3 at 6:00. Pain level on movement in the experimental and control group accordingly was – 2.0 and 3.9 at 18:00; 1.8 and 4.2 at 6:00.

Lactate level in the experimental and control group accordingly was 1.02 and 1.57 mmol/l before surgery; 1.32 and 1.08 mmol/l at 18:00; 1.9 and 1.54 mmol/l at 6.00.

Glucose level in the experimental and control group accordingly was 5.5 and 5.4 mmol/l before surgery, 8.3 and 6.4 mmol/l at 18.00; 7.5 and 5.8 mmol/l at 6.00.

**Discussion**

Dexamethasone provides excellent analgesic effects. But it does not allow the use of a lower rescue medication dose. Serum glucose and lactate rises after dexamethasone injection, but does not reach a level that affects recovery using dexamethasone in the mentioned doses and period of time.

### P26 Differential gene expression analysis as a predictor of response to neoadjuvant chemoradiation for locally advanced rectal cancer (LARC)

#### Ishtar Redman^1^, Sinead Toomey^2^, Bryan Hennessy^2^, Simon Furney^3^

##### ^1^School of Medicine, Royal College of Surgeons in Ireland, Dublin, Ireland; ^2^Department of Molecular Medicine, Royal College of Surgeons in Ireland, Dublin, Ireland; ^3^Genomic Oncology Research Group, Department of Physiology and Medical Physics, Royal College of Surgeons in Ireland, Dublin, Ireland

###### **Correspondence:** Ishtar Redman

Recent advances in the field of genomic medicine in conjunction with the advent of affordable high-throughput biological sequencing technologies have led to the accumulation of a wealth of genomic data. This project utilized RNA-sequencing techniques to elucidate differential gene expression between two cohorts of patients with locally advanced rectal cancer (LARC) who demonstrated varying responses to neoadjuvant chemoradiation. Identification of differentially expressed genes/novel pathways has the potential to inform treatment modalities, targeting patients on the basis of the molecular signatures of their tumours.

To identify differentially expressed genes transcribed in patients who responded to neoadjuvant chemoradiation. The RNA-seq counts of 17 patients with LARC who exhibited varying responses to neoadjuvant chemoradiation served as the input for this project (X responders, Y non-responders). Differential gene expression analysis was then performed using the bioconductor package *DESeq2* within *RStudio* to elucidate differences in the gene expression levels between the two response groups and to identify discriminatory genes/pathways.

Using an adjusted P-value of (0.1) and a fold change (FC) of ≤ 1.0 and ≥1.0 as cut-offs, 33 genes were found to be upregulated in the responders and 6 genes in the non-responders.

IL24, a tumour microenvironment transformer, as well as an inducer of anti-cancer immunity in murine models of colorectal cancer was upregulated (FC= 0.18) in the ‘*responders.’* The PURPL gene *(p53 upregulated regulator of p53 levels),* an intergenic long non-coding RNA (lncRNA), known to regulate basal p53 levels was also found to be upregulated in the *‘responders’* (FC = 0.044). Heparan sulfate 6-O-sulfotransferase 2 (HS6ST2) was upregulated in the *‘non-responders’*, FC = 11.178. Pre-existing literature has highlighted the over-expression of HS6ST2 mRNA in colorectal cancer and correlated its expression with poor survival outcomes. The discriminatory genes within the two patient groups may provide insights into novel therapeutic targets and challenge current treatment paradigms.

### P27 Disease acquisition in paediatric international travelers; a ten-year review at the Hospital for Sick Children

#### Stavros Lalos^1^, Shaun Morris^2^ , Michelle Science^2^, Danny Farrar^2^

##### ^1^School of Medicine, Royal College of Surgeons Ireland, Dublin, Ireland; ^2^The Hospital for Sick Children, University of Toronto, Toronto, Canada

###### **Correspondence:** Stavros Lalos

**Introduction**

International travel can expose travelers to a variety of health risks which have the potential to cause significant morbidity and mortality globally. We undertook a 10-year chart review examining imported infectious diseases associated with international travel at the Hospital for Sick Children (SickKids) in the culturally diverse city of Toronto, Canada.

**Methods**

Retrospective chart review of selected travel related diseases in children ranging in age from Birth to <18 years who were admitted at SickKids between January 1^st^, 2009 and December 31^st^, 2018. Cases were identified using an ICD-10 search of medical records. Patient demographics, travel history, epidemiological data, disease, and prophylaxis history were documented.

**Results**

In total, 156 children were diagnosed with a travel related infection and hospitalized as inpatients over a 10-year period. The most common diagnoses were typhoid or paratyphoid fever (n=58, 37%), malaria (n=57, 36%), and Hepatitis A (n=14, 8%). The median age of those infected was 8 years (IQR 3-12). There were 122 (78%) Canadian born, 31 (20%) immigrants and 3 (2%) who were visiting Canada. Of those who lived in Canada, 112 (90%) were visiting friends or relatives (VFR), 6 (5%) were traveling for tourism and 2 (2%) were travelling for humanitarian work. The most common country for acquisition of infection for Typhoid or Paratyphoid Fever was India. Most common country of infection for Malaria was Nigeria 33%. Hepatitis A was most commonly acquired in Pakistan 57%.

**Conclusion**

Imported infectious diseases continue to be a significant issue in travelers returning from trips abroad and the immigrant population. Individuals traveling to visit Friends and Relatives (VFR) remain a group that should be targeted for appropriate pre-travel advice.

### P28 Doctor’s views on ethics of childhood vaccination mandates

#### Eleni Frisira, Nick Goulding

##### Barts and the London School of Medicine and Dentistry, London, England

###### **Correspondence:** Eleni Frisira

The 2018-2019 measles outbreak has generated discussions on immunization policies and ethical dilemmas around policy design are at the center of current debates. Considering recent events, immunization policies should be re-examined. Public health strategies aim to provide the greatest benefit while minimally restricting individual liberties. This study aims to explore the views of healthcare professionals, primarily doctors, on ethics of routine childhood immunization policies, to suggest the optimal strategy for patients in England. Semi-structured interviews were conducted with nine doctors, working in primary care or paediatrics, and one specialist nurse to discuss their opinions around immunisation ethics. Immunization was described as a form of child protection but doctors were uncertain on who should define a child’s best interest in the absence of an immediate infectious threat. The contribution of parental education to improve outcomes was well accepted and issues around informed consent and coerced decision-making were raised. There were clashing considerations about the trade-off between parental autonomy and community protection due to the impact of unimmunized groups on herd immunity. The role of personal views of doctors in consulting parents on immunizations was controversial. Lastly, the impact of different strategies on patient-doctor relationships was a major concern, thinking this could affect patient care overall. With the increasing emphasis put on building participatory patient-doctor relationships and on individual liberties, mandates may not be the optimal strategy for England. However, the recent pressures demand we prioritize patient education to improve parental confidence in vaccines.

### P29 Effect of 3-day introduction of glutathione on activities of H2S-producing enzymes in kidneys under experimental nephropathy conditions

#### Kateryna Gerush, Igor Gerush

##### Bukovinian State Medical University, Chernivsti, Ukraine

###### **Correspondence:** Kateryna Gerush

Kidney diseases are accompanied by metabolic disorders and toxic lesions of the human body. H2S is mainly synthesized in kidneys and is vital for the functioning of tubular and glomerular kidney apparatus.

Prevention of development of kidney diseases by the usage of effective antioxidants for body protection from oxidative stress in cells is one of the most important problems in medicine.

There is a lot of research conducted on antioxidant properties of glutathione, but the biochemical mechanism of its influence on H2S-metabolism under nephropathy is unknown.

The experiment was conducted on albino mature rats which weighed 160 – 180 g. Nephropathy was modeled by a single intraperitoneal folic acid injection at a dose of 250 mg/kg. Animals with nephropathy got an intragastric glutathione injection for 3 days, once daily, at a dose of 100 mg/kg. Desulfurizing activity of enzymes and concentration of H2S were determined in the cortical and medullary parts of kidneys.

There was a decrease in H2S concentration in the kidneys of rats with nephropathy: in the medullary part by 27.8 % and in the cortical part by 16% compared to the control group. The activity of H2S-producing enzymes: cystathionine-β-synthase (CBS) and cysteine aminotransferase decreased by 24.3% and 30.7% respectively in cortical part of kidneys of rats with nephropathy compared to the control group*.*

3-day glutathione introduction increased the H2S-concentration in the cortical and medullary parts of the kidney by 16.8% and 24.6% respectively and increased the CBS activity by 8.3% in the cortical part of kidney.

Our results show that nephropathy causes strong disorders in H2S-metabolism. Specifically, the glutathione introduction increases H2S-concentration in kidneys. This is due to antioxidant properties of glutathione and cysteines presence in glutathione and its use in H2S- biosynthesis. We anticipate our assays to be a starting point for the next investigations of glutathione as an additional remedy for correction of kidney disease and preventing its complications.

### P30 Effect of localized radiation exposure on skeletal muscle inflammation and fibro/adipogenic progenitors in juvenile mice

#### Jessica Lloyd^1^, Donna D'souza^1^, Jillian Larkin^1^, Nicolas Collao^1^, Michael De Lisio^2^

##### ^1^School of Human Kinetics, University of Ottawa, Ottawa, Canada; ^2^Department of Cellular and Molecular Medicine, University of Ottawa, Ottawa, Canada

###### **Correspondence:** Jessica Lloyd

**Introduction**

Radiation therapy is a common treatment option for childhood cancers and has contributed to improved outcomes and increased survival rates. However, survivors have an increased risk for debilitating late-effects of radiation therapy that contribute to poor muscle health in adulthood. Radiation induced fibrosis (RIF) is characterized by muscle atrophy, fibrosis, and impaired regeneration. Fibro/adipogenic progenitors (FAPs) coordinate with inflammatory cells, including macrophages, during muscle regeneration. Previous studies have examined the effect of radiation on adult skeletal muscle; however, little is known regarding the role of FAPs and macrophages in skeletal muscle development following juvenile radiation exposure. The purpose of this study is to examine changes in FAP and muscle macrophage content over a time-course following localized radiation (IRR). We hypothesized that radiation will contribute to long-term reductions in FAP content, and a prolonged inflammatory response.

**Methods**

Five-week-old, male, CBA mice were exposed to ionizing radiation (16 Gy) to the left hindlimb (n=32). The right leg was shielded and used as a contralateral control. Mice were euthanized at 3, 7, 14, and 56 days post-IRR. Total macrophages, M1 macrophages (pro-inflammatory; F4/80^+^CD206^-^), M2 macrophages (anti-inflammatory; F4/80^+^CD206^+^), and FAPs (PDGFRα^+^) were quantified in tibialis anterior (TA) and gastrocnemius/soleus (GAS) by immunofluorescence.

**Results**

FAP content decreased at 14- and 56-days post-IRR (p<0.05) in both muscles. In the TA, total and M2 macrophage content increased 7-days (p<0.05), and M1 macrophage content decreased 7-days (p<0.05) post-IRR. In the GAS, total macrophages were increased 3-days (p<0.05), M2 macrophages were elevated at 3- and 7-days (p<0.05), and M1 macrophages were reduced at 14-days (p<0.05) post-IRR.

**Discussion**

In developing skeletal muscle, radiation decreases FAP content in late time points and promotes a temporary anti-inflammatory macrophage polarization. These data provide novel cellular targets for reducing the negative long-term consequences of radiation exposure on skeletal muscle in childhood cancer survivors.

### P31 Evolution of RULs and their impact on urogenital system in postnatal period of life

#### Maksym Horiachok, Nataliia Navarchuk

##### Higher state educational establishment of Ukraine, Bukovinian State Medical University, Chernivtsi, Ukraine

###### **Correspondence:** Maksym Horiachok

The uterus and uterine cervix ligament concept is a decisive factor for female pelvis surgery and for fundamental mechanisms of urogenital dysfunction ascertainment. The rectouterine ligament (RUL) has aroused great interest in terms of its use for surgical support in the management of pelvic organ prolapse.

The purpose of the study is to clarify the RUL macro- and microstructure in postnatal ontogenesis of different age groups.

Investigations have been performed in 37 anatomical specimens. The following investigational methods have been used: macroscopy, microscopy of histological sections series, conventional and thin preparations. Statistical data processing was performed with the licensed program “RSTUDIO.”

At the 1st, 2nd adult, elderly, and senile ages RUL consists of two symmetric peritoneal folds, which cover RUL behind uterine and laterally from the rectum. The ureter crosses lower inferior hypogastric plexus top-down, outside-inside. The hypogastric nerve is available on each side under the ureter, goes posterior-anterior, top-down, and rounds RUL from the outside. Pelvis nerves have been identified as derivatives of the third-forth anterior branches of sacral plexus, changing direction down to RUL. They join, forming the lower inferior hypogastric plexus in the lateral part of RUL and in the posterior layer of the broad ligament. In the cervix end of the RUL there is a large number of vessels available. It contains smooth muscles, dense connective tissue, blood and nerve. In the intermediate one third vessels there are a moderate number of vessels with the main tissue components being connective tissue. In the sacral ones – even less, they consist of loose connective tissue and fatty inclusions.

Our results show the RUL’s anatomical relationships, in particular, to the ureter and inferior hypogastric plexus. RUL can be subdivided into three sections and may be used for improvement of existing and development of new methods of surgical correction and treatment of different lesions of urogenital system.

### P32 Evaluation of intestinal microRNAs in newly diagnosed paediatric inflammatory bowel disease patients

#### Jyoti Dhawan^1^, Kevan Jacobsen^2^

##### ^1^School of Medicine, Royal College of Surgeons in Ireland, Dublin, Ireland; ^2^University of British Columbia, Vancouver, Canada

###### **Correspondence:** Jyoti Dhawan

**Introduction**

Inflammatory bowel disease (IBD) is a chronic relapsing gastrointestinal illness, categorized into two subtypes, Crohn’s Disease (CD) and Ulcerative Colitis (UC). In this retrospective pilot study, we will use paraffin embedded intestinal biopsy sections from newly diagnosed paediatric IBD and control patients to identify potential differences in microRNA (miRNA) expression associated with CD and UC. This project aim is to identify and quantify miRNA biomarkers that could outperform current clinical and biochemical diagnostic tests for IBD patients, and will enable a shift towards a more personalized therapeutic approach for IBD patients. Several studies in adult IBD populations have demonstrated both alterations in miRNA expression, and variations in expression at different stages of the disease. There has only been one miRNA study in the paediatric IBD population, with similar findings to the adult studies, however, the paediatric study was small and lacked a uniform population, thus the need for further research.

**Methods**

Intestinal biopsies from paediatric patients (100 CD, 100 UC, and 100 controls) will be retrospectively obtained from the pathology department, de-identified and sent for miRNA testing. The miRNA levels will be compared with multi-variable data collected from patients (including demographics, disease type, symptoms, disease activity score, type of induction and maintenance therapies across initial diagnosis, 6 month, 12 month, and 18 month periods). Inclusion criteria: IBD patients with initial diagnostic colonoscopy between January 2010 – December 2015, and controls with a normal colonoscopy with no IBD diagnosis. Exclusion Criteria: indeterminate IBD, and patients started on therapy prior to initial colonoscopy.

**Results**

Analysis and comparison of the miRNA samples with the collected data is being carried out currently.

**Discussion**

Once data analysis is complete we will be able to further understand the correlation between miRNA biomarkers and disease outcomes, as well as possible implications for tailored IBD therapy.

### P33 Exploring the barriers to use of end-tidal CO2 monitoring during in-hospital cardiac arrest

#### Nadiha Noor Chelsea^1,2^, Mr. Matthew Yang^3^, Katherine Allan^4^, Deven Chandra^5^, Natalie Wong^6^

##### ^1^The Keenan Research Summer Student Program, Department of Critical Care, St. Michael’s Hospital, Toronto, Canada; ^2^School of Medicine, Royal College of Surgeons in Ireland, Dublin, Ireland; ^3^Department of Critical Care, St. Michael’s Hospital, Toronto, Canada; ^4^Department of Cardiology, St. Michael’s Hospital, Toronto, Canada; ^5^Department of Anesthesia, St. Michael’s Hospital, Toronto, Canada; ^6^Department of Emergency Medicine, St. Michael’s Hospital, Toronto, Canada

###### **Correspondence:** Nadiha Noor Chelsea

**Background**

Monitoring end-tidal carbon dioxide (ETCO2) provides real-time data on the quality of resuscitative attempts, which is crucial for survival from in-hospital cardiac arrest (IHCA). The aim of the study was to identify barriers that responders encounter in implementing ETCO2 monitoring during IHCA events at St. Michael’s Hospital in Toronto, Canada.

**Methods**

Using purposive sampling, a total of 56 healthcare professionals including residents, anaesthesiologists, nurses and respiratory therapists were recruited to participate in a qualitative study. Six focus groups were conducted over a 9-month period (2017-2018). Interviews were tape-recorded and transcribed verbatim. Transcripts were imported into NVivo software, and then analysed using qualitative thematic analysis.

**Results**

The data suggested three principal themes of barriers to monitoring ETCO2. (1) Practical challenges: challenges existed with setting up ETCO2-equipped defibrillators. Further limitations included a lack of space and time during resuscitation, along with a shortage of team members familiar with ETCO2 equipment. (2) Questions about ETCO2 data usage: participants questioned whether ETCO2 data was incorporated into decision making during an IHCA. Lack of communication and understanding about the ETCO2 data was prevalent amongst team members and affected their motivation in recording and using the information. (3) Problems with simulation training program: participants criticized the simulation environments as being unrealistic with respect to difficulties encountered with setting up ETCO2 monitors. Simulation rooms were spacious and organized with equipped defibrillators properly arranged, whereas a real-time IHCA situation was small and chaotic. Additionally, training was only delivered during daytime to core group of staff, such that not all responders were able to undergo training prior to attending an IHCA.

**Discussion**

The study suggests that realistic modification of simulation trainings could address some of these barriers. Furthermore, training teams on ETCO2 interpretation and how to best use the data could lead to beneficial usage of ETCO2 during IHCA.

### P34 Factors influencing tRNA cleavage in FUS (1-359) ALS mouse model

#### Lisle Blackbourn^1^, Cliona Farrell^1,2^, Marion Hogg^1,2^, Jochen Prehn^1,2^

##### ^1^Department of Physiology and Medical Physics, Royal College of Surgeons In Ireland, Dublin, Ireland; ^2^FUTURE-NEURO Research Centre, Royal College of Surgeons In Ireland, Dublin, Ireland

###### **Correspondence:** Lisle Blackbourn

**Introduction**

Stress-induced transfer RNA (tRNA) cleavage occurs in systems from yeast to mammalian cells, indicating this is a highly conserved process. It has been proposed that tRNA fragments enable cells to adapt to stress. Previous work in our group has shown tRNA-derived stress-induced fragments (tiRNAs) are elevated at symptom onset (post-natal day (PND) 90), but were not elevated at presymptomatic stage (PND 50) in transgenic mice from the FUS (1- 359) mouse model. In this project, we are studying factors that influence tRNA cleavage using immunohistochemistry.

**Methods**

Spinal cords were dissected at PND 50 and 90 from wild type and transgenic littermates. Immunohistochemistry was performed using antibodies against the ribonuclease Angiogenin, its inhibitor, and using antibodies that recognise phosphorylated neurofilament (SMI-32) or Choline Acetyltransferase (ChAT) to highlight motor neuron cell bodies. Cell bodies were traced and mean expression of target antibodies within the mask were quantified. Statistical significance was assessed by a two-tailed T-test.

**Results**

Levels of angiogenin were increased for transgenic mice at onset of disease (p=0.0378), but not at presymptomatic stages. Angiogenin inhibitor was slightly decreased for transgenic mice compared to wild type mice at presymptomatic stages, whereas levels were the same around the onset of disease for transgenic and wild type mice.

**Discussion**

Angiogenin levels were significantly elevated at symptom onset whereas angiogenin inhibitor levels remained similar suggesting increased angiogenin activity may occur. Elevations of angiogenin without elevations in angiogenin inhibitor allow for increased tRNA cleavage, which could explain why we observed increased tRNA cleavage in transgenic mice at onset of disease compared to wild type, and saw no difference in levels of tRNA fragments at presymptomatic stages.

### P35 Gauging the hypolipidemic effects of nicotinic acid in patients with primary hyperlipidemia—A single blind placebo randomised controlled trial

#### Talal Almas, Muhammad Ali Niaz

##### School of Medicine, Royal College of Surgeons in Ireland, Dublin, Ireland

###### **Correspondence:** Talal Almas

**Introduction**

This study was carried out to ascertain the beneficial effects of Nicotinic acid on body weight, blood pressure, HDL-cholesterol and LDL-cholesterol in patients with primary hypertension. Although a standard pharmacological treatment regimen to treat primary hypertension persists in medical literature, the drugs used within these regimens often manifest a vast array of debilitating side effects whereas the lipid-lowering effects of Nicotinic acid remain largely unknown.

**Methods**

40 men and women with hyperlipidemia were sampled, and 20 of them were subjected to a placebo—constituting the control group—and 2.25 grams of niacin per tablet per day were given to 20 subjects over a three-month course, constituting the experimental group. The patients’ blood pressure and body weight were recorded every two weeks on follow-up, their LDL-cholesterol calculated using the Friedewald formula (LDL = TC (TG / 5 + HDL-C). The HDL-cholesterol was calculated using a direct-method serum analysis, while the triglycerides and overall cholesterol levels were calculated using a colorimetric method.

**Results**

In the experimental group , HDL-cholesterol improved from 36.41 ± 1.96 to 43.70 ± 1.81 mg / dl. Niacin reduced LDL-cholesterol from 181.98 ± 9.24 mg / dl to 118.18 ± 3.99 mg / dl, with the total percentage (%) change from 0 to 90 days being 34.67 mg / dl. Additionally, the triglycerides decreased from 170.04 ± 7.59 to 136.851 ± 6.29 mg / dl. The mean body weight also plummeted from 66.30 ± 193 kg to 64.80 ± 1.81 kg.

**Discussion**

We conclude that Niacin, when administered with the correct therapeutic dose, demonstrates a myriad of beneficial health effects such as reduced body weight and blood pressure in patients with primary hyperlipidemia. In contrast, most of the pharmacological therapy currently employed in medical practice demonstrates debilitating adverse effects that are not seen upon treatment with Niacin.

### P36 Factors influencing breast and cervical cancer screening service delivery in Malawi: A systematic review

#### Emily Panteli^1^, Chiara Pittalis^2^, Jakub Gajewski^3^

##### ^1^School of Medicine, Royal College of Surgeons in Ireland, Dublin, Ireland; ^2^Public Health and Epidemiology Division of Population Health Sciences, Royal College of Surgeons in Ireland, Dublin, Ireland; ^3^Institute of Global Surgery, Royal College of Surgeons in Ireland, Dublin, Ireland

###### **Correspondence:** Emily Panteli

**Introduction**

Cancer incidence is increasing in Malawi, with breast and cervical cancer in the top five commonest cancers among women. Outcomes are poor due to late diagnosis and limited treatment accessibility. Screening is essential to improve prognosis for these cancers. The objective of this systematic review was to determine which factors enhance or hinder the delivery of breast and cervical cancer screening in Malawi with regard to accessibility, uptake, efficiency, and impact on rates of early detection and treatment.

**Methods**

In June-August 2019 the authors searched six bibliographic databases and grey literature to retrieve relevant literature (English language only, no time or study design restrictions). Six studies were identified providing original data on breast and cervical cancer screening services in Malawi. Due to the heterogeneity of the studies regarding design and focus, a thematic analysis was conducted, using NVivo 12 and manual methods. Evidence was synthesised without formal meta-analysis.

**Results**

We identified multiple barriers influencing the delivery of breast and cervical cancer screening services in Malawi: patient factors - lack of knowledge/awareness, location, screening environment, perceived quality of care; healthcare facility factors –availability of physical/human resources, organisation of services; and healthcare system factors – inadequate funding, staffing (distribution, supervision, retention), monitoring, and lack of guidelines.

**Discussion**

While multiple interlinked barriers hinder cancer screening delivery in Malawi, the literature identified numerous useful lessons. Convenience of cancer screening, in terms of accessibility (location, opening times) and integration with other existing health services (e.g. reproductive or HIV care) has a positive effect on service uptake. Awareness of cancer and related services, and perceived quality of screening (having a dedicated room in the clinic, privacy, staff professionalism etc.) are significant determinants of patient satisfaction. Capitalising on these lessons is essential to strengthen service delivery in Malawi and improve early detection and treatment of cancer.

### P37 Haemoglobin and cholesterol affect apparent Tacrolimus clearance in paediatric transplant recipients – A retrospective cohort study

#### Elisa Yoo^1^, Guido Filler^2^, Carmen Rodriguez Cuellar^3^, Ana Catalina Alvarez-Elias^4^, Mara Medeiros^5^

##### ^1^School of Medicine, Royal College of Surgeons in Ireland, Dublin, Ireland; ^2^Western University, London, Canada; ^3^Pediatric Nephrology in Shaio Clinic Bogatá, Bogotá, Colombia; ^4^The Hospital for Sick Children, University of Toronto, Toronto, Canada; ^5^Universidad Nacional Autónoma de México, Mexico City, Mexico

###### **Correspondence:** Elisa Yoo

**Introduction**

Tacrolimus has a narrow therapeutic index with substantial inter- and intra-patient variability. Factors beyond genetic and developmental factors are poorly understood. Recent adult studies suggest that haemoglobin affects the apparent clearance (CL/F), whereas this and other potential factors in children are understudied.

**Methods**

After ethics approval, we performed a single centre retrospective cohort study of paediatric renal transplant recipients, who were followed between January 1^st^, 2004, and June 30^th^, 2018. Patients without tacrolimus therapy or concomitant sirolimus were excluded. The aim was to show the impact of haemoglobin, albumin, cholesterol and HDL on the apparent tacrolimus clearance (CL/F = Dose/AUC). Data were collected from electronic health record. We used 12-point pharmacokinetic (PK) profiles.

**Results**

Thirty-three patients were included. Median age at transplantation was 10 years, 52% were female, the median tacrolimus area under the curve (AUC) was 133 ng*h/mL. CL/F mainly correlated with haemoglobin (n=1,257, r=-0.3767, p<0.0001), HDL-cholesterol (n=236, r=-0.3973, p<0.0001) and total cholesterol (n=373, r=-0.1821, p=0.0004).

**Discussion**

The present study suggests a moderate impact of the biochemical factors studied in the tacrolimus CL/F. Lower haemoglobin seems to increase it, while higher cholesterol decreases it. Physicians should be aware of this association during the TDM follow up.

### P38 Identifying novel colorectal cancer driver genes by analyzing genome mutations

#### Razi Alalqam, Ahmad AlQalaf, Xiangmei Cui, Simon Furney

##### School of Medicine, Royal College of Surgeons in Ireland, Dublin, Ireland

###### **Correspondence:** Razi Alalqam

**Introduction**

Colorectal cancer is caused by somatic gene mutations. Defective DNA mismatch repair is one of the major causes. Microsatellite instability (MSI), which is an instability at a DNA level, has been shown to be caused by the loss of function in the DNA mismatch repair system (MMR). The identification of MSI’s has several important applications. It can be used as a marker for different tumor subtypes, and predict sensitivity to therapies and drugs. The objective of this study is to validate the genomic signatures for MSI’s associated in colorectal cancer using accurate and sophisticated computational methods.

**Methods**

Mutational catalogues were obtained from the online cancer gene database COSMIC. Rstudio was used with deconstructSigs package to analyze the mutational signatures.

**Results**

The results of this study show that signature 6, 15, 20, and 26 that are associated with defective DNA mismatch repair and found in microsatellite unstable tumors, are present in colorectal cancer gene database. Furthermore, indels were present in each sample associated with MSI signatures. Other signatures, are also present that are not associated with DNA mismatch Repair system, such as Signature 1.

**Discussion**

The mechanism behind some of the signatures remain unknown. Understanding the mechanism behind each signature will deliver a better understanding of the mutational patterns. Furthermore, correlating these mutational signatures with epidemiology and other profiles is highly needed.

### P39 Immediate changes in foot clearance patterns with Functional Electrical Stimulation (FES) and Ankle Foot Orthosis (AFO) in the presence of foot drop post-stroke

#### David Mangion, Daniel Goggi

##### Department of Physiotherapy, University of Malta, Malta, Msida

###### **Correspondence:** David Mangion

**Introduction**

Foot drop post-stroke is a significant precursor to falls to which is a substantial influencer in the quality of life. This study set out to compare any immediate changes in spatiotemporal and kinematic foot clearance patterns that occur with the use of Functional Electrical Stimulation (FES) and Ankle Foot Orthosis (AFO) in the presence of foot drop post- stroke.

**Methods**

This research adopted a case study research design. A patient, who experienced a stroke was chosen and monitored at a self-selected walking speed with a motion capture system using a Helen Hayes marker set-up without any aid, with an AFO and with FES (Bioness® L300) in the same session respectively, wearing own footwear. A Timed Up and Go (TUG) test was carried out to assess the risk of falls in all the three walking trials.

**Data Analysis**

The analysis was carried out by recording spatiotemporal values for the whole gait cycle, and kinematic data measured throughout the whole gait cycle and peak angles where interpreted for the three modalities under assessment. Recording of the TUG test after every successful set of walks in seconds for every modality was carried out, and data was then extrapolated in MS ExcelTM for analysis and compared in a same subject design method.

**Results/Discussion**

The results obtained showed inconclusive spatiotemporal values with minimal difference between AFO and FES modalities, such as 95.0 ± 2.25 steps/min to 97.6 ± 4.30 steps/min for cadence and 0.66 ± 0.051 m/s to 0.62 ± 0.032 m/s for walking speed respectively. Although FES provided less variation in movements (0.75SD) as opposed to AFO (1.2SD) and cleared the foot further off the ground by improving dorsiflexion by 2.8°, the risk of falls as interpreted by the TUG criteria increased as the results where poorer than AFO by 1.05 seconds.

### P40 Impact of SRS GammaKnife treatment on MRI-derived disease anatomy in trigeminal neuralgia patients

#### Taylor Loon^1^, Catherine Coolens^2^, Adam Dmytriw^3^

##### ^1^Royal College of Surgeons in Ireland, Dublin, Irelandl ^2^Department of Medical Physics, Princess Margaret Cancer Centre, Toronto, Canada; ^3^Department of Medical Imaging, University of Toronto, Toronto, Canada

###### **Correspondence:** Taylor Loon

**Background**

GammaKnife Stereotactic Radiosurgery (GKRS) is the favoured treatment modality in patients with refractory trigeminal neuralgia. In retreatment, it varies as to whether a new MRI scan is done for pre-planning. Examining variations in metrics of planning, radiation-induced radiological changes of anatomy, and radiation dose to critical structures can help determine if it’s beneficial for patients to undergo re-imaging.

**Methods**

We analyzed a total of 56 patients that underwent GKRS retreatment from 2012-2019 using Leksell Gamma Knife and Excel. This included radiation shot coordinates, their distance from the brainstem, thickness of the trigeminal nerve and contour, and radiation dose to the brainstem.

**Results**

The mean difference in shot X coordinates was 1.09 mm (STDV 4.98) and Y coordinates was -3.22 mm (STDV 8.8). When the horizontal distance of treatments 2’s shot was closer to the brainstem than treatment 1, the radiation dose to the brainstem was increased (COR -0.5777, p=0.0011). When the thickness of the trigeminal nerve increased, the thickness of the contour also increased (COR +0.7859, p=0.004).

**Conclusion**

Variations in treatment metrics mentioned above are considered in determining whether if it’s beneficial for TN patients to undergo an additional pre-planning session before retreatment. Further investigation into patient’s aetiology and additional radiation induced radiological changes, as well as a larger population size, and manually co-registering the images, is required to confirm whether it is beneficial to update GKRS procedure.

### P41 Investigating the impact of cisplatin resistance on neuroblastoma-derived extracellular vesicles

#### Joshua Ramjohn^1^, Thomas Frawley^2^, Olga Piskareva^2,3^

##### ^1^School of Medicine, Royal College of Surgeons in Ireland, Dublin, Ireland; ^2^School of Pharmacy and Biomolecular Sciences, Royal College of Surgeons in Ireland, Dublin, Ireland; ^3^Department of Anatomy and Regenerative Medicine, Royal College of Surgeons, Dublin, Ireland

###### **Correspondence:** Joshua Ramjohn

**Introduction**

Neuroblastoma (NB) is the most common childhood extracranial solid tumour. Chemotherapeutic resistance arises through complex genetic and epigenetic changes, and tumour-derived extracellular vesicles (EVs), such as exosomes (small EVs, sEVs), may be implicated in this process. Previous studies have shown significant upregulation of several proteins in cisplatin-resistant versus cisplatin-sensitive NB cell lines. One of these proteins, Epidermal Growth Factor Receptor (EGFR), was selected for this study due to its established role in carcinogenesis and presence in tumour-derived EVs.

Therefore, we hypothesise that changes due to cisplatin resistance in the NB cellular proteome would be reflected in the NB sEV proteome. Hence, this study aimed to determine sEV number, protein concentration and EGFR protein expression in two NB cell lines, Kelly (cisplatin-sensitive) and KellyCis83 (cisplatin-resistant).

**Methods**

Two human-derived NB cell lines, Kelly and KellyCis83, were cultured. EVs were isolated by differential ultracentrifugation, characterised by Nanoparticle Tracking Analysis (NTA), western blots and Transmission Electron Microscopy (TEM), and then quantified by NTA. This was followed by protein quantification of sEV proteins and western blots to estimate EGFR expression levels in EVs and cell lysates (CL).

**Results**

There was no significant difference in the sEV number and protein concentration between the Kelly and KellyCis83 cells. However, there was a six-fold increase in EGFR expression in KellyCis83 sEVs compared to Kelly sEVs.

**Discussion**

This study suggests that cisplatin resistance may result in sEV enrichment of EGFR, since it alters the sEV expression of EGFR, without altering sEV number and protein concentration. This indicates that changes due to cisplatin resistance in the cellular proteome may be reflected in sEV proteome. Future studies, involving mass spectrometry of sEVs from both cell lines, and cell proliferation, viability, migration and colony forming assays, may succour in determining the potential of EGFR as a putative prognostic marker in NB.

### P42 Investigating the use of transcutaneous bilirubin measurements on neonates with unexposed areas of skin to phototherapy

#### Maria Casalino^1^, Michael Sgro^2^, Thivia Jegathesan^3^

##### ^1^School of Medicine, Royal College of Surgeons in Ireland, Dublin, Ireland; ^2^St. Michael’s Hospital, Toronto, Canada; ^3^University of Toronto, Toronto, Canada

###### **Correspondence:** Maria Casalino

**Background**

Transcutaneous bilirubin measurements (TCB) are currently used as a screening tool prior to phototherapy to determine at-risk infants for hyperbilirubinemia. Once a baby undergoes phototherapy, or exceeds the threshold for treatment, only blood samples measuring total serum bilirubin (TSB) are used to monitor hyperbilirubinemia. When an infant is under phototherapy TSB is measured every 6-12 hours, which endues much stress and pain on the neonate. The use of TCB after phototherapy commences would be beneficial – however most studies have reported deceased effectiveness as a result of exposed skin under phototherapy. Only some pilot studies have studied the effectiveness of TCB on unexposed areas to phototherapy by covering the skin. These are limited to small sample sizes.

**Methods**

A prospective cohort study of neonates > 35 weeks receiving phototherapy at St. Michael’s Hospital. At the time of each clinically required TSB, two TCBs will be completed within 15 minutes. The TCBs will be recorded on exposed and unexposed areas of the forehead during phototherapy. Once a neonate starts phototherapy a 3M RedDot electrode will be used to cover a part of the forehead during phototherapy. To determine the impact of covering the skin during phototherapy, agreement and correlation coefficients, as well as Bland-Altman Plotting, between TCB and TSB will be completed.

**Results**

129 TCB measurements have been conducted on neonates > 35 weeks gestation on exposed areas of skin to phototherapy resulted in a mean difference of -16.9 umol/L (-25.7 to -8.1). To determine the effects of covering the skin, 200 term infants undergoing phototherapy will be recruited and TCB values on covered areas of skin will be evaluated at the time of a TSB

**Conclusion**

Covering the skin may offer a new approach to using TCB after phototherapy.

### P43 Is there a benefit to the lowest possible CA125: The relationship between biomarkers and survival in epithelial ovarian cancer

#### Leen Alhoussan^1^, Mitchell Clark^2^, Marcus Bernardini^2^

##### ^1^School of Medicine, Royal College of Surgeons in Ireland, Dublin, Ireland; ^2^University of Toronto, Toronto, Canada

###### **Correspondence:** Leen Alhoussan

**Introduction**

To investigate the relationship between CA125 levels at diagnosis, post-operatively and completion of treatment in ovarian cancer in relation to progression free survival.

**Methods**

Retrospective cohort study at the UHN of women diagnosed with ovarian cancer between January 2008-December 2015 with complete medical records. All CA125 levels will be collected during up-front treatment as well as important clinical and pathologic variables. Survival analysis will be completed by Cox proportional hazard models and Kaplan-Meir methods. CA125 levels within the normal range will be divided into <5, 6-10 and 11-35 IU. Progression free survival (PFS) is defined as the time from completion of primary treatment to date of first recurrence.

**Results**

349 patients met eligibility criteria. Among patients treated with primary cytoreductive surgery (PCS), CA125 at completion of treatment is significantly associated with PFS (HR 1.3, p=0.013) however, CA125 at completion of treatment is not associated with PFS in neoadjuvant chemotherapy (NACT) (p=0.053). In PCS, the difference between pre-operative and post-operative CA125 is significantly associated with PFS (HR1.25, p<0.001) but not in NACT (p=0.397). There is no statistically significant difference in PFS between women who have a CA125 of <5IU, 6-10 IU or 11-35 IU at the completion of primary treatment (p=0.218). In PCS, women who receive more than 6 cycles of chemotherapy have a worse PFS (10.6 months) vs. those who receive 6 (35.8 months p=0.018). In NACT, there is no PFS advantage to >3 cycles of chemotherapy.

**Discussion**

The differences in CA125 before and after treatment are important in assessing a woman’s risk for progression after PCS and adjuvant chemotherapy, but not in NACT. Achieving the lowest possible level of CA125 within the normal range was not associated with a survival advantage. Additional chemotherapy beyond the standard regimen does not improve survival and leads to further toxicity.

### P44 Is use of anticholinergic medication associated with poorer cognitive performance in Mild Cognitive Impairment (MCI)?

#### Githmi Palahepitiya Gamage^1^, Adam Dyer^2^, Cathy McHale^2^, Joshi Dookhy^2^, Deborah Fitzhenry^2^, Desmond O'Neill^2^, Sean P Kennelly^2^

##### ^1^School of Medicine, Royal College of Surgeons in Ireland, Dublin, Ireland; ^2^Memory Assessment Clinic Tallaght University Hospital, Dublin, Ireland

###### **Correspondence:** Githmi Palahepitiya Gamage

**Introduction**

Anticholinergic medication use is associated with an increased risk of cognitive impairment and dementia. Less clear is the evidence concerning anticholinergic use in those with Mild Cognitive Impairment (MCI), who may represent a particularly vulnerable group.

**Methods**

Participants with a confirmed diagnosis of MCI on the basis of history, neuropsychological evaluation, and neuroimaging were included. Medication lists were obtained from participants and the number of medications with potential/definite anticholinergic properties was calculated assigning an Anticholinergic Cognitive Burden (ACB) Score to each participant. Cognitive function was assessed using the Mini-Mental State Examination (MMSE) and the comprehensive multi-domain Repeatable Battery for the Assessment of Neuropsychological Status (R-BANS).

**Results**

105 participants were included (mean age: 74.5 ± 5.3; 53/105, 50.4% female). Over two-fifths (46/105, 43.8%) were prescribed a medication with potential/definite anticholinergic properties and a small minority (8/105, 7.6%) had a high total ACB Score (>3). There was no difference in MMSE score between those using an anticholinergic medication vs non-users (26.3 vs 26.5, t = -0.17, p = 0.57). Similarly, there were no differences in scores on the Immediate Memory (t = -1.03, p = 0.15), Visuospatial/Constructional (t = 0.01, p = 0.49), Language (t = -0.93, p = 0.17) or Attention (t = -0.09, p = 0.46) domains of the R-BANS. No difference was seen for any domain in those with a high total ACB score.

**Conclusion**

We observed a high burden of anticholinergic medication use in those with MCI. However, this was not associated with poorer cognitive performance, which may be due to limited study power given our cohort only demonstrated subtle impairment. Longitudinal follow- up of this cohort will assess whether anticholinergic medications influence progression of MCI to dementia and its potential as a modifiable risk factor.

### P45 Occurrence of hypertension in school-going adolescents based on the fourth report and AAP guidelines: A comparative study

#### Pooja Rangwala^1^, Yash Thakker^1^, Radhe Shah^2^, Ayush Thakkar^3^, Dhairy Upadhyay^1^, Gurusharan Dumra^1^

##### ^1^AMC MET Medical College, Ahmedabad, India; ^2^Smt. NHL Municipal Medical College, Ahmedabad, India; ^3^GCS Medical College, Hospital and Research Centre, Ahmedabad, India

###### **Correspondence:** Pooja Rangwala

**Introduction**

Hypertension has found increased incidence globally, especially in school-going population. Aim of the study is to find out and compare the occurrence of hypertension amongst school-going adolescents based on the “Fourth Report on the Diagnosis, Evaluation, and Treatment of High Blood Pressure in Children and Adolescents” (2004) and the “American Academy of Pediatrics (AAP) Clinical Practice Guideline for Screening and Management of High Blood Pressure in Children and Adolescents” (2017). Also, to look for any correlation of hypertension in these children with their Body Mass Index (BMI), diet, exercise and family history of hypertension.

**Methods**

A cross-sectional study was carried out on adolescents attending different schools during June-August 2019. Their age, weight and height were measured. Blood pressure (BP) was measured on 3 consecutive days and classified using the Fourth Report and AAP Clinical Guidelines. Revised IAP Growth Charts for Height, Weight and Body Mass Index for 5- to 18-year-old Indian Children were used to classify the BMI of children.

**Results**

The sample size consisted of 258 students between the age-group of 10-16 years. According to the AAP guideline, 51(19.77%), 11(4.26%) and 4(1.55%) had hypertension stage-1, hypertension stage-2 and elevated BP respectively; as compared to the Fourth Report, according to which 41(15.89%), 7(2.71) and 3(1.16%) were hypertension stage-1 & 2 and pre-hypertension respectively. Chi-square test showed a strong correlation between obesity and hypertension (p-value is 0.000359) in these children.

**Discussion**

According to the AAP guidelines, hypertension stage-1 & 2 and elevated BP was found to be 1.24, 1.57 and 1.33 times more respectively as compared to the Fourth Report. Also, obesity plays a large role in the development of adolescent hypertension. Early detection of elevated blood pressure and associated mild obesity or overweight can lead to prevention of its evolution into full-blown disease as the child matures.

### P46 Patients’ preferences for psychiatric models of care

#### Anna Sophia Cacciola^1^, Jalen Manett^2^, Weam Sieffien^3^, Gili Adler Nevo^3^

##### ^1^School of Medicine, Royal College of Surgeons in Ireland, Dublin, Ireland; ^2^Wilfrid Laurier University, Waterloo, Canada; ^3^University of Toronto, Toronto, Canada

###### **Correspondence:** Anna Sophia Cacciola

Although humans have always suffered from detriments to mental health, psychiatry as the modern profession it is known as today, is relatively new. Psychiatry is a field in which models of care are numerous and consistently emerging. Both psychotherapy and biological psychiatry are continuously evolving. We are currently able to provide a multitude of medications for varied diagnoses, as well as an abundance of psychotherapy modalities. Psychotherapy is currently on the decline in psychiatry, while pharmacotherapy and other biological interventions dominate. All interventions provided by psychiatrists are evidence based and well researched, but rarely have patients been asked for their preference and its influence on the effectiveness of their care. This study aims to bridge that gap by asking patients about their preferred interventions. The main objective of this study is to determine the alignment between patients’ preferences and available resources for the management of mental health conditions. Our hypothesis is that psychiatric intervention, regardless of modality, is more effective when it is the patients’ treatment of choice. At the moment, there are currently no results available for this study, however, this study aims to survey 50 patients who are currently accessing mental health services at Michael Garron Hospital and WoodGreen Community Services in Toronto, Ontario, Canada. The exclusion criteria would include: Patients with psychotic disorders and learning disorders, non-English speakers and patients under the age of 18. It is believed that by offering a patient their treatment of choice, they will be more inclined to be engaged in their treatment and less likely to withdraw as a result of their engagement.

### P47 Proposed smartphone application to improve adherence to hydroxyurea among Irish adolescents and young adults with sickle cell disease

#### Saif Ullah Syed^1^, Helen Fogarty^2^, Alan Gaul^1^, Natalija Aleksejenko^1^, Maniya Chowdhary^1^, Róisín Daly^1^, Emmanuel Eguare^1^, Kate MacNamara^1^, Anthony Maher^1^, Oliseyelum Ojo^1^, R Geoghegan^2^, H Conroy^2^, N Ngwenya^2^, E Crampton^2^, E Tuohy^2^, C McMahon^2^

##### ^1^School of Medicine, Royal College of Surgeons in Ireland, Dublin, Ireland; ^2^Haematology Department, Crumlin Children’s Hospital, Dublin, Ireland

###### **Correspondence:** Saif Ullah Syed

**Introduction**

Sickle cell disease(SCD) is an autosomal recessive disorder, characterized by chronic haemolytic anaemia, vaso-occlusive complications and end-organ damage with declines in health-related quality of life(HRQOL) domains. Hydroxyurea has demonstrated significant advantages in terms of morbidity and mortality in SCD, however, medication non-adherence is a major issue, especially in the teenage and adult population. To counteract this, we investigated the usefulness of a mobile phone application and its features that would improve adherence in an Irish SCD context.

**Methods**

We administered an anonymous cross-sectional survey to SCD patients regarding hydroxyurea attending St James’s Hospital and Children’s Health Ireland at Crumlin, Dublin. Medication adherence measures included components of the Modified Morisky Adherence Scale(MMAS) and a visual analogue scale. We also designed the features and business-model required for the successful launch of an app based on patient preferences.

**Results**

63 patients responded in total: 63% female and 37% male(median age 17 years) and 76% average compliance rate using a visual analogue scale. The greatest barrier to adherence was forgetfulness with 67% requiring family reminders for their medication. 87% were interested in an app to promote increased adherence, with the most popular proposed features including daily medication reminders(73%), adherence progress tracking(77%) and facts about SCD(72%). Based on thorough market analysis we estimated cost of app development and launch to be approximately €37,375 for android and €39,150 for iOS platforms, respectively.

**Discussion**

Although medication adherence rates were relatively high in our survey, however, we acknowledge that patient self-report may overestimate compliance. Furthermore, forgetfulness was a common theme throughout the survey domains and interest in an app to promote improved adherence to hydroxyurea was high. Forming over 10% of the Irish SCD population, our survey provides novel and valuable insights into medication adherence as well as unique features needed in any future app targeting this problem.

### P48 Maternal diet during lactation and allergy in infants: A systematic review and meta-analysis

#### Anna Berbenyuk^1^, Daria Levina^1^, Aysylu Gamirova^1^, Daniel Munblit^2^

##### ^1^Sechenov University, Moscow, Russia; ^2^Imperial College London, London, England

###### **Correspondence:** Anna Berbenyuk

**Introduction**

Food allergy is a major public health concern affecting around 1 in 20 infants, with high costs to public health services. Breast milk is the main source of food proteins for infants during the first six months of life. Physicians often recommend dietary restrictions to the mothers during lactation to reduce the risk of allergy development. The aim of this research is to identify associations between maternal diet and allergic diseases development in later life.

**Methods**

We performed a search in MEDLINE and EMBASE databases. 4484 papers published between inception and 2019 were found. Three researchers selected relevant studies and extracted data independently. Thirteen RCT studies met inclusion criteria and full-text review and data extraction were performed. Quantitative analysis included random-effects meta- analyses using inverse variance method.

**Results**

Dietary intervention was presented by maternal avoidance of allergenic products during lactation period only. Subgroup meta-analysis was performed separately for two age groups (0-2 years and 4-10 years) at the age of health outcome assessment. Data from four studies on children aged 0-2 suggests that maternal food avoidance can reduce the risk of eczema development (Odds Ratio [OR] 0.52; 95% CI 0.32, 0.85). Maternal dietary restrictions did not result in a significant decrease of other allergic disease (asthma, sensitisation, food intolerance and rhinoconjunctivitis) risk. In three RCTs investigating long-term health outcomes (4-10 years), dietary interventions during lactation showed protection against rates of sensitization (OR 0.40; 95% CI 0.25, 0.63) but did not impact any allergic diseases development.

**Conclusion**

Our findings suggest that there is still no strong evidence that maternal diet during lactation influences infants’ allergy incidence. The reduced risk of eczema seen in the early age group was not seen in children of an older age group. Methodological bias and heterogeneity in health outcome definition and result reporting do not allow for definitive conclusions.

### P49 Measuring brown adipose tissue and its relationship to metabolic health using infrared thermography in 8-10 year old males

#### Emily Hutchings^1^, Frank Ong^2^, Basma Ahmed^3^, Elizabeth Gunn^2^, Stephan Oreskovich^2^, Gregory Steinberg^3^, Katherine Morrison^2^

##### ^1^School of Medicine, Royal College of Surgeons in Ireland, Dublin, Ireland; ^2^Department of Pediatrics, McMaster University, Hamilton, Canada; ^3^Department of Biochemistry and Biomedical Sciences, McMaster University, Hamilton, Canada

###### **Correspondence:** Emily Hutchings

**Introduction**

Brown adipose tissue (BAT) is a thermogenic tissue, induced by cold, and maybe a therapeutic target for obesity and diabetes. The purpose of this study is to investigate the use of infrared thermography (IRT) as a method to detect BAT and to relate IRT BAT measures to metabolic health in children.

**Methods**

The study population consisted of n=18, males with a mean age of 9.74 ± 0.93 years. Dexa scan and fasting blood draws were obtained. The supraclavicular (SCV) and acromion (control) temperature were assessed using IRT before and after a 1-hour 18^0^C cold exposure. SCV temperature pre (TSCV) and post-cooling (Δ TSCV, Δ TSCV-Acromion, post-cold) were used as measures of BAT. Paired sample T-tests compared mean SCV temperature pre and post- cooling, mean SCV to mean acromion temperatures and compared differences in BAT measurements on the left and right side. Pearson’s correlation for normally distributed data and Spearman’s Correlation coefficient for non-normally distributed data were used to assess correlations between variables.

**Results**

An increase in temperature post-cooling was measured in the left (p= 0.001), and right (p=0.029) SCV regions. TSCV was negatively correlated with non-HDL cholesterol r= -0.537, p=0.018 and body fat percentage r=-0.812, p= <0.001. TSCV was positively correlated with HDL cholesterol r=0.518, p=0.023. Δ TSCV was negatively correlated with fasting glucose r=-0.622 p= 0.01.

**Discussion**

An increase in SCV temperature following cooling is measurable using IRT in children and may represent BAT activity. Limitations to measuring BAT via IRT include overlying subcutaneous fat and regional blood flow, which may influence heat emission. Comparing the acromion to the SCV temperature may be an effective method for controlling against adiposity and blood flow. Overall, correlations between adiposity, glucose, cholesterol, and BAT may demonstrate that low BAT activity is associated with the development of obesity and associated metabolic disturbances.

### P50 Method of microbial biofilms destruction In chronic wounds

#### Iryna Kozlovska

##### Department of Surgery, Higher state educational establishment of Ukraine, Bukovinian State Medical University, Chernivtsi, Ukraine

**Introduction**

Сhronic wounds (CW) is caused by microorganisms in biofilm. The aim was to develop a method of destroying biofilms by electrophoresis and to determine the optimal current density, which would have the best bactericidal effect on the bacteria and destroy the microbial biofilms.

**Materials & Methods**

The species and populations of microflora in 144 CW and microorganisms’ ability to form biofilms were studied. We determined biofilm density and optimal current density which destroy biofilm.

**Results**

Bacteria isolated in monoculture (E. coli and Ps. аeruginosa) in 100% cases formed high density biofilm. Bacteria in monoculture exhibit stronger adhesive properties and their biofilms matrix was denser, which better protects them from antimicrobial medicines. Mixed bacteria formed high-density biofilms - from 50.0% to 83.3%, medium density – 16.9%-50.3%, low density – 10.1%-13.8%.

The action of direct current electric field with the density of 0,025 mА/cm^2^ did not have a bactericidal effect on cells in the biofilm, although the density of the latter decreased by an average of 1.5 times. With increase of electric density to 0,05-0,1 mА/cm^2^ the biofilm matrix was destroyed more intensively, its density decreased from high to middle and low. This led to the death of bacteria, which caused their decrease in the destroyed biofilm from 10.7 to 56.4 times (p <0.05).

**Conclusion**

The formation of microbial biofilms complicates antimicrobial therapy and determines the chronic nature of the wound process. So treatment of CW should include not only antibiotic therapy, but also new methods of influencing biofilms of appropriate density. In complex treatment of CW we recommend conducting electrophoresis with a current density of 0,05-0,1 mA/cm², and antibacterial therapy should be designed with the previously investigated sensitivity of microbes from biofilms isolated from chronic wounds.

### P51 Neuroprotective properties of gastropeptide Pro-Gly-Pro and its acetylated form in the conditions of glutamate excitotoxicity

#### Arina Zgodova^1^, Daniil Frolov^2^, Zanda Bakaeva^3^

##### ^1^Sechenov University, Moscow, Russia; ^2^MIREA, Russian Technological University, Moscow, Russia; ^3^National Medical Research Center for Children’s Health, Moscow, Russia

###### **Correspondence:** Arina Zgodova

**Introduction**

Glutamate (Glu) is one of the most common excitatory neurotransmitters which excess of can cause a pronounced excitotoxicity. Pro-Gly-Pro peptide (PGP) effectively protects the gastric mucosa from damage caused by prolonged administration of monosodium glutamate (food supplement). PGP also is able to influence the structure of the central nervous system. Its acetylation makes PGP more resistant to the action of prolyl peptidases and prevents its hydrolysis. The purpose of this research was to investigate the effect of PGP and AcPGP on the survival of a neuroglial culture under glutamate excitotoxicity.

**Materials and methods**

Primary neuroglial cell cultures were obtained from 1-day old Wistar rat cortexes, grown under standard conditions. On day 11-12, PGP and AcPGP 10 μM was added 1 hour prior to glutamate exposure 33 μM. The neuron survival was assessed by the ratio of living/dead cells after 24 hours. The survival of cultured neurons was determined using the vital fluorescent dyes (Syto-13 for living cells, EthD-1 for dead cells).

**Results**

PGP 10 μM and AcPGP 10 μM are not neurotoxic. Glu 33 μM reduced the ratio of living/dead cells, and its excitotoxicity effect was 45%. PGP increased the number of living cells after its incubating with Glu (27%). Acetylation of the peptide obstructed the manifestation of the effect under study. The difference between the groups Glu and Glu+AcPGP was 6%.

**Conclusion**

PGP can weaken the excitotoxic effect of Glu 33 μM on cultured cortical neurons and has the neuroprotective effect peptide in given concentration. AcPGP does not affect neuron survival. It can be assumed that the acetylation of the peptide at the N-terminus reduces the ligand-receptor interaction and prevents the manifestation of its neuroprotective effect. Therefore, it is possible that PGP has a gastro-neuroprotective effect, which could be mediated by its dipeptide derivative, GP.

### P52 Neurotransmitter release and synaptic vesicles endocytosis in calcium ringer

#### Georgii Krivoshein, Pavel N Grigoryev, Andrey L Zefirov

##### Kazan State Medical University, Department of Physiology, Kazan, Russia

###### **Correspondence:** Georgii Krivoshein

Endocytosis, exocytosis and vesicular transport are basic steps of synaptic vesicle cycling. However, not all mechanisms of these processes are yet completely understood.

The aim of the present work was to investigate how the process of neurotransmitter release and synaptic vesicles endocytosis work under a significant increase in extracellular Calcium ion concentration.

Optical method: Fluorescence confocal microscopy with the fluorescent dye FM 1-43 (6uM) and electrophysiological method - intracellular recording of postsynaptic potentials were used. To depolarize motor nerve endings a method of electrotonic depolarization using a glass extracellular microelectrode was practiced (B. Katz, 1969). Experiments were performed on neuromuscular preparations of frog's cutaneous-pectoris muscles. Standard Ringer’s solution and calcium Ringer’s solution (containing in mM: CaCl2 83, KCl 2.5, NaHCO3 2.2, pH 7.2-7.4) were taken. Pre-terminal portions of nerve fibers were stimulated with a single impulse duration of 4 ms in the presence of 4-Aminopyridine (1 mM). To prevent muscle fibers contraction in standard Ringer’s solution tetrodotoxin (1 mM) was added.

In standard Ringer’s solution, the mean amplitude of miniature endplate potentials (mEPPs) was evaluated to 1.2±0.36 mV (n=5). In calcium Ringer’s solution, a distinguished decline in the amplitude of mEPPs to 0.26±0.14 mV (n=5) was revealed. The frequency (Hz) of mEPPs was increased twice from 1.5±0.45 in the standard Ringer’s solution to 3.0±0.56 in calcium’s Ringer solution (n=5). The mean amplitude of evoked endplate potentials was higher in calcium’s Ringer solution – 50±7.1 mV (n=5), than in standard Ringer’s solution – 25±4.8 mV (n=5). When stimulation in the presence of the fluorescent dye was applied, bright round fluorescent spots in motor nerve terminals were found.

After isotonic replacement of sodium ions by calcium in the extracellular solution, considerable electrophysiological changes in neurotransmitter release and the presence of synaptic vesicles endocytosis were noticed.

### P53 Non-invasive pre-clinical atherosclerosis detection in patients with refractory hypertension in South India

#### Varahabhatla Vamsi^1^, Mitalee Garg^2^, Basavaprabhu Achappa^3^, Padmanabh Kamath^4^, Vaman Kulkarni^5^, Ingrid Prkacin^6^

##### ^1^Zaporizhzhiya State Medical University, Zaporizhzhiya, Ukraine; ^2^Kasturba Medical College, Mangalore, India; ^3^Department of Medicine, Kasturba Medical College, Affiliated to MAHE, Mangalore, India; ^4^Department of Cardiology, Kasturba Medical College, Affiliated to MAHE, Mangalore, India; ^5^Department of Community Medicine, Kasturba Medical College, Affiliated to MAHE, Mangalore, India; ^6^Department of Internal Medicine, School of Medicine, University of Zagreb, Zagreb, Croatia

###### **Correspondence:** Varahabhatla Vamsi

The endothelium is a complex endocrine organ regulating haemostasis, maintains vascular tone, vessel diameter, platelet-leukocyte vascular adhesion, vascular permeability and tissue growth and regulation. Vessel tone alteration is an important factor for pre-clinical atherosclerosis (pcA) development. Refractory hypertension (rHTN) is clinically established if BP remained uncontrolled after ≥3 visits to a clinic within a minimum 6‐month follow‐up period. Novel non-invasive oscillometric devices assessing pulse wave velocity(PWV) could possibly detect pcA in hypertensive patients.

To detect pcA in patients with rHTN using non invasive oscillometric device and establishing correlation with PWV and central blood pressure (CBP).

We assessed the values of CBP, PWV, Brachial systolic (sys) and diastolic (dys), peripheral (PPp) and central pulse pressures (PPc), stroke volume (SV), Cardiac output (CO), mean arterial pressure (MAP), total peripheral vascular resistance (TPVR), Heart rate (HR), Augmentation index (AIx) in 200 patients. 148 Male (M) subjects and 52 female (F) subjects with refractory hypertension using noninvasive Agedio B900 device (Germany) at Cardiology department, KMC Mangalore were included.

The mean age of 200 patients was 57,35±13,38; 57,33±13,5 in M; 57,40±12,38 in F. BP(sys) in M was 141,1±21,3; 141,3±20,7 in F respectively. The CBP values in M was recorded to be 130,1±20,8 and 128,5±20,7 in F respectively. The values of SV, TPVR, HR in M were 71,6±14,1; 1,3±0,2; 76,18±13,7 and 67,1±16,2; 1,49±1,22; 77,5±13,4 in F respectively. The MAP, PPp, PPc and AIx values in M were 115,6±15,2; 47,44±14,4; 34,12±10,3 and 112,46±17; 51,82±15,2; 38,7±11,9; 29,4±13 in F respectively. AIx in M was 20,94±13,9 and 29,4±13,2 in F respectively. The difference between sexes was statistically significant for PWV (M/F: 8.8/8.6 m/s p<0,01).

Non-invasive PWV and AIx are the surrogate markers for assessing endothelial dysfunction and arterial stiffness. Rise in vascular stiffness correlates with pre-clinical atherosclerosis formation inside the vessels of patients with rHTN.

### P54 Organization of psychology programs within pediatric hospitals

#### Katerina Green^1^, Emily Hernandez^2^, Aoife Reilly^1^, Carisa Parrish^3^

##### ^1^Royal College of Surgeons in Ireland, Dublin, Ireland; ^2^California State University Northridge, Los Angeles, California, United States of America ^3^Johns Hopkins Medical Institution, Baltimore, United States of America

###### **Correspondence:** Katerina Green

**Introduction**

As US hospitals move from quantity-based fee-for service models to quality-incentivized alternative payment models, the cost-effectiveness of behavioural interventions has become more evident. Literature has not yet analyzed the effectiveness of organizational structures of these programs within hospital settings and this has created a problem for psychologists to advocate for changes and support within their departments and hospitals. The purpose of this study was to summarize pediatric psychology within children’s hospitals ( ie. organizational structure, psychologist:bed ratio).

**Methods**

A convenience sample (n=21) of the best Children’s hospitals in the US, was selected after review of the Children’s Hospitals ranked in the 2019-2020 US World and News Report^1^. Methods employed included online reviews of hospital websites and the Association of Psychology Postdoctoral and Internship Centers (APPIC), email correspondence with directors or leadership in hospital pediatric psychology and follow-up phone calls. Data on hospital characteristics was gathered and descriptive statistics calculated.

**Results**

Ratios of psychologists to inpatient beds varied considerably (minimum of 3.1:1 to maximum of 45.88:1). The most common organization of pediatric psychology programs was a program within departments of psychiatry and/or behavioural sciences. 41% reported having a psychology director. Only 18% had a separate division of pediatric psychology.

**Discussion**

The results suggest that psychologists are often found within related departments of psychiatry and behavioural science. This raises the question of whether psychologists are represented and supported within their departments. Results also indicated wide variability in psychologist ratio to inpatient beds. This study is ongoing and will eventually summarize the different organizations of psychology programs and psychologists within pediatric hospitals to support growth of these divisions.

A limitation of this study was the variable reliability of information on organization and demographics of psychologists within hospitals and need to verify with each program.

### P55 Outcomes of endoscopic treatment for plantar fasciitis: A systematic review

#### Leona Ward^1^, Yoshiharu Shimozono^2^, John G. Kennedy^2^

##### ^1^Royal College of Surgeons in Ireland, Dublin, Ireland; ^2^Department of Orthopaedic Surgery, NYU Langone Health, New York, USA

###### **Correspondence:** Leona Ward

**Background**

Endoscopic plantar fascia release (EPFR) is an established surgical treatment for recalcitrant plantar fasciitis. Few studies assess the mid to long-term outcomes of this procedure making it difficult for surgeons and patients to establish realistic postoperative expectations. The purpose of this systematic review is to provide a comprehensive review on the outcomes of endoscopic plantar fascia release in the treatment of plantar fasciitis at mid- and long-term follow up.

**Methods**

A systematic review was performed using MEDLINE, EMBASE and Cochrane library databases in March 2018 based on the Preferred Reporting Items for Systematic Reviews and Meta-Analyses (PRISMA) guidelines.[1] Studies included were evaluated with regards to level of evidence (LOE) and quality of evidence (QOE) using the Coleman methodological score.

Clinical outcomes and complications were also evaluated.

**Results**

Twenty-one studies including 601 feet were included in this systematic review with a mean follow up of 26.9 months. 16 papers used the American Orthopaedic Foot and Ankle Society (AOFAS) score. The weighted mean preoperative AOFAS score was 52.3 and the postoperative score was 88.75 out of 100. The total number of patients who had complications was 111 of 601 (18.7%). The most common complication was recurrence of pain experienced by 7.49% of patients (45 of 111).

**Conclusion**

Endoscopic plantar fascia release provides good clinical and functional outcomes in patients with refractory plantar fasciitis. This procedure is associated with a moderately high complication rate. However, heterogeneous study designs and low level and quality of evidence limit the current literature. As a result, further well designed studies are necessary to determine the optimal treatment for refractory chronic plantar fasciitis.

### P56 Pathological diagnosis of cutaneous squamous cell carcinoma in Constanta County Hospital, Romania

#### Nesa Samuel Karunadhas, Madalina Bosoteanu

##### Clinical Service of Pathology, Emergency County Hospital “Sf. Apostol Andrei” Constanța, Faculty of Medicine, University “Ovidius” of Constanța, Constanta, Romania

###### **Correspondence:** Nesa Samuel Karunadhas

**Introduction**

Cutaneous squamous cell carcinoma (cSCC) is the second most common skin malignancy, often associated with chronic exposure to ultraviolet rays (UV). The study aimed to ascertain the number of patients diagnosed with cSCC and its subtypes comprising various skin lesion excisions sent to the Department of Histopathology in Constanta County Hospital, Romania.

**Methods**

A retrospective study was conducted from 2014 to 2018. Each patient’s medical number, date of admission, initials, age, sex, pathological diagnosis, grading and staging were analysed. Conventional histopathological methods with hematoxylin-eosin stain and immunohistochemical evaluation were effectuated.

**Results**

The study involving (n-702) patients consisted of 340 (48.4%) males (M) and 362 (51.5%) females (F). 2015 had the most number of diagnoses,169 (24.07%). The youngest patient was 11 years old, diagnosed with seborrheic keratosis. The oldest patient was 96 years old, diagnosed with keratinised cSCC. 141 (20%) patients were diagnosed with cSCC. 116 (16.5%) patients were diagnosed with keratinised cSCC, 69 (M), 47 (F). 10 (1.42%) patients were diagnosed with non-Keratinised cSCC, 5 (M), 5 (F). 7 (0.9%) patients were diagnosed with Verrucous cSCC, 5 (M), 2 (F). 7 (0.9%) patients were diagnosed with Acantholytic cSCC, 6 (M), 1 (F). 1 (0.1%) (F) patient was diagnosed with Basaloid cSCC. 42 (5.9%) patients were diagnosed with Actinic Keratosis (AK),18 (M), 24 (F). 105 (14.9%) patients were diagnosed with Keratoacanthoma, 46 (M), 59 (F).

**Discussion**

There was a higher prevalence rate for keratinised cSCC. A constant rise in AK rates was observed and a similar trend was perceived for keratoacanthoma. Untreated AK has a 20% prospect of progression to cSCC. These inclinations could be correlated to the upsurge in smoking rates and chronic exposure to UV rays without adequate protection or the application of tanning beds among the Romanian population.

### P57 Prevention of the development of reperfusion complications in the treatment of atherosclerotic multilevel occlusions of the main arteries of lower

#### Kyrylo Pantsiuk^1^, Iryna Kozlovska^2^, Oleksandr Kolotylo^2^

##### ^1^Higher state educational establishment of Ukraine, Bukovinian State Medical University, Chernivtsi, Ukraine; ^2^Department of Surgery, Higher state educational establishment of Ukraine Bukovinian State Medical University, Chernivtsi, Ukraine

###### **Correspondence:** Kyrylo Pantsiuk

One of the causes of the adverse effects of reconstruction of the aorto-hip-popliteal segment is the development of reperfusion complications.

The aim of the work is to prevent the development of reperfusion complications through the use of techniques and surgical interventions in the revascularizing surgical treatment of atherosclerotic multilevel occlusion of the major arteries of the lower extremities.

46 patients with a high risk of reperfusion complications were included in the work. In all patients, the lower extremity with a high risk of reperfusion complications was characterized by III A-B–IV сhronic arterial insufficiency (CAI), the contralateral lower limb – II A-B CAI. To determine the degree of ischemic lesions of the lower limb, a modified classification of Fontaine R. has been applied taking into account the criteria of the European Working Group (1992).

Aortic decliрation determines the course of blood flow restoration along the main arteries of the lower extremities. In aorto-bifemoral bypass, blood flow should be restored along the branch of bypass alternately. The blood flow is restored first along the branches of the unproblematic lower limb. Secondarily, blood flow is restored along the branches of the lower limb, from which reperfusion complications are expected. The indicated sequence of restoration of blood flow according to alloprosthesis makes it possible to halve the force of the shock wave of blood flow along the arterial bed of the problem lower limb.

In case of multilevel atherosclerotic occlusal process of the lower extremity arterial bed: aorto/iliac-femoral and femur-popliteal segments, in which there is a suspicion of reperfusion complications, only aorto/ilio-femoral bypass is suspected. The specified volume of the operation makes it possible to eliminate the propagation of the shock wave of blood flow in the distal segments of the lower limb.

### P58 Prognostic Importance of Synthropy of Congenital Malformations of Nervous System in Children

#### Irina Lastivka, Volodymyr Davydiuk

##### Department of Pediatrics and Medical Genetics, Higher State Educational Institution, Bukovinian State Medical University, Chernivtsi, Ukraine

###### **Correspondence:** Irina Lastivka

**Introduction**

Congenital malformations of the central nervous system, especially as a part of multiple congenital malformations, occupy a leading position in the structure of infant mortality, morbidity and primary childhood disability. Their timely diagnosis is a priority in pediatrics and pediatric surgery.

**Methods**

For the purpose of studying the prognostic value of the synthropy of congenital malformations of the central nervous system, we analysed cases of multiple congenital malformations that included congenital malformations of the central nervous system. We divided the cases into Group I, (based on the Regional Medical-Genetic Center of Chernivtsi register in 2000-2019, and Group II, 45 lethal cases of multiple congenital malformations (based on the material of autopsies of the regional pathological and anatomical bureau of Chernivtsi, in the same years).

**Results**

According to the results of the study, in both groups the most common anomaly was congenital musculoskeletal defects, the second - congenital heart defects, the third - congenital malformations of the urinary system. The incidence of congenital heart defects in the multiple congenital malformations of group II significantly exceeded that of group I (p <0.001), which could have influenced the prognosis for the life of the child. In addition, group II showed a number of birth defects (pulmonary hypoplasia, biliary atresia, congenital bowel obstruction), which did not occur in group I and could also increase the risk of mortality in children.

**Discussion**

Congenital heart defects can determine the prognosis in children with multiple congenital malformations, including congenital malformations of the central nervous system. Congenital heart defects detected antenatally by ultrasound can serve as markers for the presence of multiple birth defects in the fetus and have diagnostic and prognostic value. This is of practical importance for choosing the tactics of childbirth and surgical tactics in the treatment of this category of children.

### P59 Prognostic value of modified MELD score in double valve replacement

#### Grace Kwok^1^, Yu Yu Juan^2^, Yiu Kai Hang^2^

##### ^1^School of Medicine, Royal College of Surgeons in Ireland, Dublin, Ireland; ^2^University of Hong Kong, Hong Kong, China

###### **Correspondence:** Grace Kwok

**Introduction**

Double valve replacement (DVR) is a standard surgical procedure for mitral and aortic valve disease, which has been safely performed in elderly population in the recent years. Although DVR has shown to have satisfactory outcome in short term, mortality in the long term remains high.

The Model of End Stage Liver Disease excluding International Normalized Ratio (MELD-XI) and the modified Model of End Stage Liver Disease replacing International Normalized Ratio with albumin (MELD-Albumin) has been reported as predictors for adverse events in liver disease, heart disease, and surgery. However, its predictor value in patients undergoing double valve replacement is not well understood.

**Methods**

A total of 262 patients (mean age, 59.8 years; men 93, women 169) underwent DVR between 2007 and 2019 were evaluated. 88% of patients were diagnosed with chronic rheumatic heart disease. Baseline clinical, laboratory, and echocardiographic parameters were collected in all patients, with laboratory test collected at intervals (pre 1 month, post 1 month, and post 1 year operation). Adverse outcome was defined as heart failure and all-caused death.

**Results**

The preliminary results showed that MELD-XI and MELD-Albumin score were good predictors of 1-year adverse outcomes (area under the curve: 0.65 and 0.71, respectively). Kaplan-Meier survival curve suggested that high score of MELD-XI (≥12.0) and MELD-Albumin (≥10.7) were associated with an increase risk of adverse events.

**Discussion**

Both MELD-XI and MELD-Albumin score can be used as a prognostic measurement for patients undergoing double valve replacement surgery to prevent the occurrence of adverse events. Early detection and treatment of high MELD-XI and MELD-Albumin score before DVR may reduce the occurrence of adverse events. This project is still ongoing, and further study with larger population size will demonstrate stronger significance of modified MELD score used as a prognostic measure for DVR.

### P60 Rare genetic variants associated with lung cancer in a Romanian population

#### Alexandru-Ioan Pîntea^1^, Cristina Constantin^2^, Ana-Maria Gheorghe^1^, Silvia-Ioana Andrei^1^, Dan-Denis Boloca^1^, Dan-Denis Boloca^1^, Elena Poenaru^1^

##### ^1^Carol Davila University of Medicine and Pharmacy, Bucharest, Romania; ^2^Fundeni Clinical Institute, Bucharest, Romania

###### **Correspondence:** Alexandru-Ioan Pîntea

**Introduction**

For more than a decade, the genome-wide associations studies (GWAS) represented a step forward in the identification of genetic risk factors for cancer. Today, the GWAS continue to reveal new single nucleotide polymorphisms (SNPs) associated with lung cancer. Lung cancer is a major public health problem in Romania and other Eastern European countries, with incidence and mortality rates among the highest worldwide.

**Methods**

The study included a cohort of genotyped Romanian subjects. 1835 lung cancer cases and 1437 cancer-free controls passed a data filtration process and were included in an association test between lung cancer and 95205 genetic markers.

**Results**

Nine tested variants reached GWAS significance (p-value < 5 x 10^-8^) and 82 SNPs had a p-value under 5 x 10^-6^. Among the most prominent SNPs are rs10508047 (p-value = 2.44 x 10^-11^, odds ratio = 0.26) and rs4445762 (p-value = 1.85 x 10^-8^, odds ratio = 0.58), both on the NALCN-AS1 gene, rs6558165 (p-value = 3.42 x 10^-8^, odds ratio = 0.47) on the CCAR2 gene (which is proven to have a suppressing role in lung cancer) and rs2284985 (p-value = 1.19 x 10^-8^, odds ratio = 0.57), on the SIM2 gene.The SNPs mentioned above have not been previously associated with lung cancer. They are protective variants and have a lower allele frequency in cases than in controls. Also, their allele frequency in cases is lower than in the world and European populations.

**Discussion**

Our study, the first GWAS of lung cancer patients in Romania, shows a convincing correlation between this pathology and genetic variants that have not been previously known to have any clinical significance. These findings are important, given the high incidence and mortality rates of lung cancer in Romania.

### P61 Refining the role of actomyosin contractility for platelet function

#### Reema Alsufyani, Ingmar Schoen

##### Royal College of Surgeons in Ireland, Dublin, Ireland

###### **Correspondence:** Reema Alsufyani

**Introduction**

Platelets mediate haemostasis. Platelet adhesion, aggregation and clot contraction requires contractile forces mediated by myosin IIA pulling on actin filaments. Myosin IIA mutations can cause MYH9-related disease resulting in bleeding. To which extent myosin dysfunction compromises platelet functions beyond platelet biomechanics, namely the secretion of alpha granules, is not well understood. By directly inhibiting myosin IIA *in vitro* using blebbistatin (BBT) we aimed to distinguish between effects on the actin cytoskeleton and granule secretion, respectively.

**Methods**

Blood was obtained from healthy consenting volunteers according to national regulations. Washed platelets were seeded onto fibrinogen (Fg) or von Willebrand Factor (vWF) coated coverslips in the presence of BBT (1-100μM) for 1 hour, fixed, stained for filamentous actin and vinculin, and imaged by confocal microscopy to determine the alignment of actin fibers. To address platelet secretion, washed platelets were preincubated with BBT (1.25...80 μM) and anti-P-selectin for 10 minutes, then activated with 10 nM thrombin receptor activating peptide (TRAP) and analysed by flow cytometry to determine the % of P-selectin positive platelets.

**Results**

BBT concentrations 16 μM and higher inhibited actin stress fiber formation of spread platelets on Fg, as well as on vWF. P-selectin expression in TRAP-activated platelets decreased between 0-10 μM BBT by 20% and remained constant at higher doses.

**Discussion**

The sensitivity of actin morphology to BBT on vWF and Fg indicates that platelet contractility defects affect both, adhesion and aggregation steps. The partial reduction of P- selectin expression by BBT shows that granule secretion is controlled by contractility-dependent and independent pathways.

### P62 Sample size determination in qualitative research

#### Maryam Ehtesham^1^, Tom O'Connor^2^

##### ^1^Royal College of Surgeons in Ireland, Dublin, Ireland; ^2^School of Nursing & Midwifery, Royal College of Surgeons in Ireland, Dublin, Ireland

###### **Correspondence:** Maryam Ehtesham

**Introduction**

Qualitative research is used in a variety of fields to elicit information that cannot be quantified. The challenges that the mismatch between the philosophical basis and practical methods of qualitative research poses remain an area of ambiguity. Since qualitative research does not work towards a numerical outcome, the application of statistical techniques to obtain the optimal sample size remains unsuccessful. Thus, sample size determination for qualitative studies is a challenge that researchers have tried to combat by means of different strategies.

**Methods**

Using a systematic review methodology, top ranked journals in Scopus in the fields of pharmacy, nursing, medicine and physiotherapy were searched for qualitative research papers published in 2018. Data relating to sample sizes and justification for sample sizes were extracted from purely qualitative studies with mixed methods research being an exclusion criteria.

**Results**

Data was extracted from 38 papers which met the inclusion criteria. These were broken down across the disciplines as pharmacy 39%, nursing 39%, medicine 11% and physiotherapy 11%. The review demonstrated that the justifications offered were similar across the board. They were normally either due to saturation or generally had a pragmatic basis involved. In a significant proportion of cases there was no justification offered for the sample sizes employed, demonstrating a lack of transparency. It was also noted that sample sizes in qualitative studies were relatively small generally. The mean and standard deviation were 40 and 52 respectively. The primary justification was saturation followed by no justification.

**Discussion**

It is concluded that the research offers insight on a gap in knowledge in qualitative research in the field of healthcare. It addresses the limitations in regards to sample size as well as the reasons governing such. The importance for increased sample size reporting by qualitative researchers in addition to larger sample sizes is concluded.

### P63 Synoptic operative reporting for thyroidectomies: A quality improvement project

#### Angela Mazzuchin^1^, Albino Chiodo^2^, Antoine Eskander^2^, Bradley Hubbard^2^, William El Masri^2^

##### ^1^Royal College of Surgeons in Ireland, Dublin, Ireland; ^2^Department of Otolaryngology-Head & Neck Surgery, Michael Garron Hospital, Toronto, Canada

###### **Correspondence:** Angela Mazzuchin

**Introduction**

In an era where communication has become instantaneous, medicine has lagged behind, particularly when it comes to dictated post-operative reports by surgeons. The aim of this project was to choose a new type of post-operative report that would result in superior communication of thyroidectomy procedures. Based on an initial literature review, it was hypothesized that a synoptic operative report would improve how surgical reports were created.

**Methods**

To discover which elements of an operative report were most important, a number of sources were consulted. Guidelines for the creation of synoptic operative reports, put forward by the American Thyroid Association (ATA) and Canadian Partnerships Against Cancer (CPAC), were compared to existing thyroidectomy synoptic reports from other hospitals in North America and current operative report templates. Data elements that appeared consistently across reports were deemed important, and further input from the surgeons at Michael Garron Hospital was used to determine relevant information for the department.

**Results**

Upon completion it was deemed necessary to design two operative forms for thyroidectomies, one for procedures including lateral neck dissections, the other without. These forms included 59 and 54 data elements respectively, the difference being owed to the variations in the procedures. The report was then added to charts as a PowerNote in the electronic medical records system used at Michael Garron Hospital.

**Discussion**

As an ongoing project, the reports are currently being evaluated for completeness, efficiency, and physician satisfaction. In the future there is the potential to analyze hospital savings, point of care patient outcomes, contributions to resident education, and the reports validity to contribute to future databases.

### P64 Shear stress strains cognition: Linking cerebral blood flow and cognitive performance

#### Kalle Amolins^1^, Ian Newhouse^2^, Kurt Smith^2^

##### ^1^School of Medicine, College of Surgeons in Ireland, Dublin, Ireland; ^2^School of Kinesiology, Lakehead University, Thunder Bay, Canada

###### **Correspondence:** Kalle Amolins

**Introduction**

Engaging in acute bouts of sub-maximal aerobic exercise increases cerebral blood flow (CBF). Concomitant increases in CBF and intra-arterial shear stress (SS) mechanically trigger a cascade of endothelial markers known to enhance cognitive performance. In contrast, it is unknown if acute reductions in CBF and SS will attenuate cognitive performance. This study therefore aimed to quantify the impact of acute elevations and reductions in CBF and SS on cognitive performance in otherwise healthy adults.

**Methods**

Participants (n = 11; ♂ = 5; ♀ = 6; 18-35 years) each performed cycling exercise (≥120 bpm) and hyperventilatory hypocapnia (-10 mmHg PetCO2) interventions to increase or decrease CBF and SS for 15 minutes respectively. Transcranial colour duplex sonography was used to measure intra-arterial diameter (MCA_D_), velocity (MCA_V_), blood flow (MCA_Q_), and shear stress (MCA_SS_) in the middle cerebral artery (MCA) prior to, during, and following each intervention. Cognitive performance was assessed prior to and following each intervention. Primary experimental outcomes were compared to baseline using a two-tailed paired samples t-test and assessed for bivariate correlation strength using a two-tailed Pearson test.

**Results**

Exercise increased MCA_D_ (Δd = 8.87%; SD = 7.75%), MCA_Q_ (ΔQ=44.30%; SD=27.68%) and MCA_SS_ (ΔSS=11.42%; SD=12.02%) during and following exercise. Hyperventilation reduced MCA_D_ (Δd=-9.88%; SD=5.47%), MCA_Q_ (ΔQ=-39.47%; SD=13.62%) and MCA_SS_ (ΔSS=-17.89%; SD=16.43%) during and following hyperventilation. Exercise did not enhance cognitive performance as compared to baseline, whereas cognitive performance was reduced following hyperventilation as compared to baseline and exercise.

**Conclusions**

This study was the first to both measure MCA_Q_ and MCA_D_ during hypocapnia and dynamic exercise and demonstrate an attenuation in cognitive performance following acutely reduced MCA_Q_ and MCA_SS_ in healthy humans. This latter finding may highlight the potential importance of acute endothelial stimulation in the maintenance of cognitive health.

### P65 Systematic review on the prevalence of incontinence-associated dermatitis in acute healthcare settings

#### Abdulaziz Alghanam^1^, Declan Patton^2^, Zena Moore^2^

##### ^1^School of Medicine, Royal College of Surgeons in Ireland, Dublin, Ireland; ^2^School of Nursing & Midwifery, Royal College of Surgeons in Ireland, Dublin, Ireland

###### **Correspondence:** Abdulaziz Alghanam

**Introduction**

IAD is a skin condition characterized by skin inflammation of the perineal area due to urine or stool exposure. It is mostly common among incontinent, elderly patients and requires special care. The aim of this systematic review was to measure the prevalence of incontinence-associated dermatitis (IAD) in acute care settings among adult patients.

**Methods**

A systematic review, following PRISMA guidelines, was conducted across three databases (CINAHL, Medline, and Embase) in search of published articles on the prevalence of IAD in acute hospitals.

**Results**

From the 7 included studies, the prevalence of IAD among all adult patients in acute care ranged from 2.7% to 28.7%. The prevalence of IAD among incontinent adult patients in acute care ranged from 29% to 45.7%.

**Discussion**

This review demonstrates that the prevalence of IAD among adult patients in acute settings varies greatly due to numerous factors. The review also found that studies measuring IAD prevalence have different criteria for IAD assessment and use different surveys to collect IAD data from patients. Therefore, there is a necessity to establish standardized IAD assessment guidelines and valid IAD prevalence surveys in order to enhance the accurate measurement of IAD prevalence in acute hospitals.

### P66The effectiveness of aspirin post-paediatric cardiac surgery

#### Noor Alkhaleefa^1^, Irene Regan^2^

##### ^1^School of Medicine, Royal College of Surgeons in Ireland, Dublin, Ireland; ^2^Our Lady's Children's Hospital, Crumlin, Dublin, Ireland

###### **Correspondence:** Noor Alkhaleefa

**Introduction**

Children with congenital heart disease often undergo high-risk cardiac surgical procedures. The routine use of aspirin is given to children post cardiac surgery in order to prevent platelet aggregation. 2-5mg/kg is prescribed empirically. There are no prospective studies that prove the effectiveness or adequateness of aspirin’s antiplatelet effect in children after congenital heart surgery. Therefore, is 2-5 mg/kg of aspirin effectively inhibiting platelet aggregation post cardiac surgery in children?

**Methods**

Retrospective review of 65 cardiology charts from 2015-2018 at Our Lady's Children's Hospital, Crumlin. Phone calls were made to ask parents about children's compliance to aspirin drug type and method of distribution of drug. Laboratory results were given blindly and used to determine the percentage of platelet inhibition.

**Results**

23.08% or 15 children on an aspirin dose of 1.13-1.28 mg/kg received adequate and effective treatment. 76.92% or 50 children were inadequately treated. Most children were on a 2-5 mg/kg dose of aspirin and underwent Glenn, Fontan, and Norwood procedures. All ages and both genders were present throughout which did not impact results. All patients were compliant to aspirin according to phone calls to parents.

**Discussion**

2-5 mg/kg of aspirin is not fully effective in bringing about treatment in some patients. Factors that affect treatment include: dosing method-aspirin type, genetic mutations, age- metabolism rate, surgery type, and rate of absorption. Further work needs to be done to rule out the above factors.

### P67 The potential role of GRP78 as a biomarker in poor prognostic colorectal cancer subgroups

#### Shivaali Karelia, Robbie Carson, Hajrah Khawaja, Sandra Van Schaeybroeck

##### Queen’s University, Belfast, Northern Ireland

###### **Correspondence:** Shivaali Karelia

**Introduction**

In order to improve colorectal cancer (CRC) patient management, the Consensus Molecular Subtypes (CMS) and CRC Intrinsic Subtypes (CRIS) were developed to stratify patients based on the genomic makeup of their tumours. There is a need for new clinical biomarkers in CRC to help identify poor prognostic patient subgroups, in need of novel treatments. Glucose regulated protein 78 (GRP78), encoded by the *HSPA5* gene, is a molecular chaperone implicated in the survival of multiple cancers. The aim of this project was to determine whether GRP78 has a potential role as a biomarker in CRC.

**Methods**

CRC clinical datasets (GSE39582/GSE103479), obtained from the NCBI Gene Expression Omnibus repository, were analysed for *HSPA5* expression and its impact on patient overall survival (OS). GRP78 levels were quantified in fresh frozen primary CRC tumour samples using Western blotting (ethics approval number: 19.12v1). Figures and statistical analysis were generated using GraphPad Prism 5 software.

**Results**

Western blotting analysis revealed that GRP78 expression was markedly higher in patient’s CRC tumours compared to their normal matched tissue. *HSPA5* expression was significantly increased in CMS1/CRIS-A subtypes of CRC (one-way ANOVA: p<0.0001***) and in proximal *BRAF* mutant tumours (p<0.0001***). High *HSPA5* expression levels were associated with a worse OS in stage 2 CRC patients.

**Discussion**

GRP78 was highly expressed in CMS1/CRIS-A subtypes of CRC, both which are associated with a high frequency of *BRAF* mutant tumours, higher histopathological grade and worse survival post relapse. In accordance, GRP78 may have clinical relevance as a biomarker for poor prognostic CRC.

### P68 The scale of diagnosis of appendicitis in children aged 15-17 years

#### Daryia Palityka

##### Grodno State Medical University, Grodno, Belarus

**Introduction**

Diagnosis of acute appendicitis (AA) in children is an actual problem of surgery. Current trends in the diagnosis of this disease suggest the following main tasks: early and accurate detection of patients requiring emergency surgery, reducing the number of complications of AA, reducing the number of negative appendectomies. To that end, diagnostic scales of AA were proposed. One of the most common diagnostic scales is the Pediatric Appendicitis Score (PAS). As it was demonstrated by us in our studies earlier, the PAS cannot be recommended for use in children 15–17 years old.

**Aim**

To develop a scale for the diagnosis of AA in children aged 15–17 years.

**Methods**

22 signs were analyzed. At the first stage, an analysis of the informativeness of the signs of Kulback was performed. Based on the analysis, diagnostic coefficients (DC) were calculated for signs with informativeness more than 1.0. The values ​​of the signs were replaced by DC. At the second stage, discriminant analysis was performed and the SDA (Scale of Diagnosis of Appendicitis) equation was obtained.

**Result**

There was developed SDA, which includes 6 signs (pulse rate, leukocyte count, ESR, sex, DC of muscle tension, DC of Right Lower Quadrant pain to cough/percussion/ hopping) and a constant.The accuracy of the coincidence of the clinical diagnosis and the predicted diagnosis was 95.7% (CI 91.5 - 99.8). When cross-checking identified 92.4% (CI 87.0 - 97.8) observations.

**Discussion**

Diagnosis of AA in children should be accompanied by regular analysis of the results and be constantly corrected depending on the opportunities of the hospital. An analysis of the results of application of SDA, depending on age, is necessary.

### P69 What elements are important for trust in the patient pharmacist relationship? A scoping review

#### Thamer Alsharif^1^, Jephin Joseph^1^, Erica Smyth^2^, Teresa Pawlikowska^2^

##### ^1^School of Medicine, Royal College of Surgeons in Ireland, Dublin, Ireland; ^2^Health Professions Education Centre RCSI, Dublin, Ireland

###### **Correspondence:** Thamer Alsharif

**Introduction**

Pharmacists’ extended role is developing from medicines experts to patient- centred professionals. A strong relationship between patients and the pharmacist has shown to improve patient outcomes and reduce healthcare costs. Trust is known to be an important aspect of the relationship between healthcare providers and patients. It also is linked to patient enablement. The gap in the knowledge base regarding trust between patients and pharmacists is lacking. The justification for this review stems from this. The role of trust in the pharmacist and patient relationship can be explored with a scoping review: existing evidence on the nature of trust and its importance can be mapped out.

**Aim**

Exploring the elements of trust in the pharmacist and patient relationship using a scoping review approach.

**Methods**

Scoping review process followed Arksey and O’Malley framework. Databases used: EMBASE, WEB OF SCIENCE, CINAHL, Psych INFO and PUBMED. A team of two (JJ and TA) independently screened title and abstracts. Relevant full texts were again assessed for eligibility by each team member. Disagreements were resolved by ES/TP. Finally, data extraction was carried out to map out some of the elements under investigation.

**Results**

From our review, we found 12 elements of trust between pharmacists and patients: perception, familiarity, friendliness, communication-skills, advice, privacy and confidentiality, professionalism, cultural competence, benevolence, knowledge and information source, technical competence and pharmacist constitutional factor

**Discussion**

This scoping review helped define the importance of trust in the pharmacist-patient relationship in different contexts as well as the elements that shape it. These findings can inform future research in pharmacy practice and education.

### P70 Understanding the contribution of P2X7 receptor in the pathology of neonatal seizures

#### Razi Alalqam^1^, Jonathon Smith^1^, Annette Nicke^2^, Tobias Engel^1^

##### ^1^Royal College of Surgeons in Ireland, Dublin, Ireland; ^2^University of Munich, Munich, Germany

###### **Correspondence:** Razi Alalqam

**Introduction**

The risk of having a seizure is greatest at the neonatal period. It can lead to various disabilities such as epilepsy, autism and death. P2X7 receptor is an ATP-gated ion receptor located on the membrane of cells. It is mainly activated by high amounts of extracellular released ATP occurring during pathological conditions, thus P2X7 targeting may lead to less side effects. This study aims to further understand P2X7 receptor in neonatal seizures to reach a possible therapeutic approach.

**Methods**

The model used was seizures induced mouse pups (p7) via intraperitoneal injection of kainic acid (KA). Four groups were included: Transgenic (Tg) KA, Wild-type (Wt) KA, Tg PBS, Wt PBS. Brains were collected 72hrs post KA and prepared for immunofluorescence double staining. Analysis of cells was carried out on the hippocampus and cortex using confocal microscopy. Three regions in the hippocampus were considered, the dentate gyrus, CA3 and CA1. Similarly in the cortex, Cx1, Cx2 and Cx3 regions. To support our results, Western blots were carried to identify the expression of P2X7.

**Results**

Upon microglia analysis, there was an increased cell count in Tg KA compared to Wt PBS. Also, colocalization of P2X7 with Microglia was detected in both transgenic groups. Contrarily, oligodendrocyte count showed no trend. No observed colocalization was detected of P2X7 with oligodendrocytes, astrocytes or neuronal beta tubulin. Furthermore, western blots representation showed increased P2X7 expression in the hippocampus of Tg KA.

**Discussion**

This study shows P2X7 contribution to neonatal seizures following KA injection. Analysis of this model at different time points post-KA injection would yield a better understanding of P2X7 function. Future studies are needed to observe P2X7 in neonates using other forms of insults such as hypoxia-induced injury.

### P71 Vascular risk factor burden in individuals with mild cognitive impairment: An opportunity to prevent progression to promote brain health?

#### Githmi Palahepitiya Gamage^1^, Adam Dyer^2^, Cathy McHale^2^, Joshi Dookhy^2^, Deborah Fitzhenry^2^, Desmond O'Neill^2^, Sean P Kennelly^2^

##### ^1^School of Medicine, Royal College of Surgeons in Ireland, Dublin, Ireland; ^2^Memory Assessment Clinic, Tallaght University Hospital, Dublin, Ireland

###### **Correspondence:** Githmi Palahepitiya Gamage

**Introduction**

Recent research suggests that over 30% of one’s lifetime risk of developing dementia is attributable to modifiable lifestyle and vascular risk factors (VRFs). These VRFs include alcohol intake, smoking, elevated BMI, hypertension and diabetes. On foot of previous evidence suggesting that management of VRFs may delay the onset of dementia, we aimed to assess the burden of VRFs in individuals presenting to a memory clinic with Mild Cognitive Impairment (MCI), who may be an increased risk of progression to dementia.

**Methods**

Participants who had a confirmed diagnosis of MCI on the basis of history, neuropsychological evaluation, and neuroimaging were included. Participants were asked about known VRFs and a comprehensive medical history was obtained. Blood pressure and Body Mass Index (BMI) were measured in a standard fashion.

**Results**

We reviewed 129 patients who were diagnosed with MCI in our memory clinic. Mean age was 74.5 (± 5.32) and 65 (50.4%) were female. Nearly one-fifth (22/116, 19.0%) reported excessive alcohol intake (>21 units/week) and nearly two-fifths (39/117, 39.3%) were current or ex-smokers. A history of Ischaemic Heart Disease was noted in 23% (27/117) and just under 10% (12/123, 9.8%) reported a history of stroke. Of those with BMI measured, 61.8% (47/76) had a BMI of 25 or greater. Two-fifths (39.8%, 49/123) had a history of hypertension.

**Conclusion**

We report a significant burden of VRFs in individuals presenting to a memory clinic with MCI. Increasing knowledge around the burden of such VRFs in cognitively vulnerable individuals in addition to optimal management of same may produce cognitive benefit in this high risk group.

### P72 Willingness of medical students to refer patients to a physician associate or a doctor based on clinical scenarios when time is a trade-off

#### Saif Ullah Syed, Pauline Joyce, Richard Arnett

##### School of Medicine, Royal College of Surgeons in Ireland, Dublin, Ireland

###### **Correspondence:** Saif Ullah Syed

**Introduction**

The Physician Associate (PA) role was piloted in Dublin, Ireland between 2015 and 2017. The concept of a PA and the acceptance of the role has been previously explored from the perspective of the patient. How medical student’s previous experience and knowledge influences their referral decisions to the emerging role of PAs as future interns remains uncertain, and needs to be explored before expanding the role in Ireland.

**Methods**

A quantitative study was undertaken using an online survey with a sample of 1909 medical students. Based on 3 medical scenarios, they were asked to choose a referral to a PA or a doctor, with 6 time trade-off options offered. Year of study, country of residence and previous experience of working/treatment with PAs and Nurse Practitioners were recorded. Descriptive statistics and Rasch analysis were used.

**Results**

177 medical students (undergraduate and graduate entry) took part in the survey. No significant difference was found between the groups. Students from countries with established PA roles and those that had previous experience with them choose the PAs more often. Overall PAs were chosen where wait time was shorter. Doctors were chosen where the time difference was less.

**Discussion**

This study confirms that medical students’ willingness to refer a patient is influenced by past familiarity with the PA role and severity of the medical condition. In addition, wait time is a primary motivator. These findings suggest a need to improve communication about the PA role among medical students to build confidence among future medical colleagues.

